# Adaptive Cu Reconstruction in Heterostructure Drives High‐Rate Nitrate‐to‐Ammonia Conversion

**DOI:** 10.1002/advs.76573

**Published:** 2026-07-13

**Authors:** Chunyu Yuan, Saikat Bolar, Yongzheng Zhang, Akitaka Ito, Tatsuhiko Ohto, Takeshi Fujita

**Affiliations:** ^1^ School of Engineering Science Kochi University of Technology Kami City Kochi Japan; ^2^ Carbon Neutral Materials Center Kochi University of Technology Kami City Kochi Japan; ^3^ Quantum Science Center of Guangdong‐Hong Kong‐Macao Greater Bay Area Shenzhen China; ^4^ Department of Physics Southern University of Science and Technology Shenzhen China; ^5^ Graduate School of Engineering Nagoya University Nagoya Aichi Japan

**Keywords:** adaptive reconstruction, heterostructure engineering, nitrate reduction reaction

## Abstract

Dynamic structural and phase evolution commonly occur during electrochemical nitrate (NO_3_^−^) reduction, leading to the formation of catalytically favorable active phases, particularly in mixed‐valence metal species. However, large overpotentials and the accumulation of undesired byproducts still severely limit the efficiency of NH_3_ synthesis. In this study, starting from a CuO/CoO_X_ (Co_1_Cu_9_O_X_) pre‐catalyst, an adaptively reconstructed heterojunction (R‐Co_1_Cu_9_O_X_) with mainly Cu/CoO_X_ is rationally engineered, synergistically catalyzing NO_3_^−^RR involving multiple intermediates. At −0.2 V vs. RHE, optimized R‐Co_1_Cu_9_O_X_ delivers an ammonia yield of 54.68 mg h^−1^ mg_cat_^−1^ with a Faradaic efficiency of 95.40%. Structural analyses reveal that Cu species undergo a self‐adaptive reconstruction equilibrium involving a surface‐localized hydroxylated oxidized Cu species coupled with reduced metallic Cu domains. Such adaptive evolution promotes nitrate adsorption and stabilizes key nitrogen‐containing intermediates. Meanwhile, the dual‐phase heterogeneous interface optimizes interfacial *H supply, ensuring precise regulation of the adsorption and hydrogenation of nitrogen‐containing intermediates. This heterostructure engineering with self‐adaptive reconstruction offers a promising strategy for advanced NO_3_^−^RR catalysis under dynamic changes in the chemical state of Cu.

## Graphical abstract

An adaptively reconstructed Cu/CoO_X_ heterojunction, featuring a surface‐localized, hydroxyl‐rich oxidized Cu species, forms in situ from a CuO/CoO_X_ (Co_1_Cu_9_O_X_) pre‐catalyst during electrochemical nitrate reduction. The self‐adaptive reconstruction and the incorporation of heterostructure regulate nitrogen‐containing intermediate adsorption and *H supply, enabling synergistic catalysis with high ammonia yield and Faradaic efficiency at low overpotential.

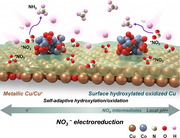

## 1 Introduction

Electrocatalytic nitrate reduction to ammonia has emerged as a promising alternative for advancing sustainable green energy under mild conditions [1, 2, 3]. Additionally, NO_3_^−^, as a ubiquitous water contaminant originating from agricultural fertilizer runoff and industrial waste, can be directly mitigated through the nitrate reduction reaction (NO_3_^−^RR) to generate NH_3_ and help restore the imbalance in the global nitrogen cycle.

Cu, as widely recognized promising candidate catalyst for advancing electrocatalytic NO_3_^−^RR, exhibits close alignment between its *d*‐band energy levels and the LUMO 𝜋* orbital of NO_3_^−^, enabling electron acceptance from high‐energy NO_3_^−^ orbitals and back‐donation into the NO_3_^−^ LUMO 𝜋* orbital, thereby achieving optimal nitrate adsorption energy and favorable kinetics that promote the initial reduction of NO_3_^−^ to nitrite (NO_2_^−^) [4, 5]. Additionally, the ability of Cu to coexist in multiple chemical states (Cu(II), Cu(I), and Cu(0)) endows it with electrochemical transformability, while intrinsic reconstruction and surface modifications induced by external stimuli allow the catalyst to dynamically adapt to evolving reaction conditions [6, 7, 8]. The dynamically evolved mixed‐valence Cu species contribute to the adsorption and stabilization of key intermediates during NO_3_^−^RR, while simultaneously mitigating competitive hydrogen generation [9, 10, 11]. However, accurately tracking the dynamic redox equilibrium and unambiguously identifying reconstruction behavior remain insufficiently understood.

Furthermore, the weak adsorption of NO_3_^−^RR intermediates (such as *NO_2_) on Cu, along with the kinetic barrier arising from its poor H_2_O dissociation ability, particularly at low overpotentials, impedes the subsequent hydrogenation steps essential for efficient NH_3_ production [12, 13, 14]. In heterogeneous catalysts, introducing secondary components into Cu can modify the electronic and geometric properties of single Cu‐based catalysts, leading to enhanced charge transfer and a more favorable *H supply for ammonia generation [15, 16, 17, 18]. These components may serve as synergistic promoters by providing additional active sites through interfacial electronic interactions and improving stability under reducing conditions [9, 19].

This study presents a straightforward and effective approach for synthesizing CuO‐based pre‐catalysts through the incorporation of secondary metals (Fe, Co, Ni, Ru, or In) using a rapid chemical oxidation method under ambient conditions. Among all the designed pre‐catalysts, the Co_1_Cu_9_O_X_ catalyst exhibits relatively superior NO_3_^−^RR performance. Quasi‐in‐situ X‐ray photoelectron spectroscopy (XPS) and transmission electron microscopy (TEM) analyses of reconstructed heterojunction with mainly Cu/CoO_X_ (R‐Co_1_Cu_9_O_X_) reveal that Cu species undergo adaptive kinetic reconstruction involving a surface Cu–OH‐rich oxidized Cu species coupled with reduced metallic Cu domains over extended reaction periods in response to the catalytic environment rather than following a simple unidirectional reduction pathway. Furthermore, operando EPR, in‐situ Raman, and attenuated total reflection surface‐enhanced infrared absorption spectroscopy (ATR‐SEIRAS) measurements, along with density functional theory (DFT) calculations, collectively demonstrate that self‐adaptively reconstructed Cu related sites mainly serve as the active centers for the initial NO_3_^−^ adsorption and activation, while the incorporated CoO_X_ sites provide a sufficient supply of *H for the deep hydrogenation of N‐containing intermediates. This study underscores the critical role of heterostructure engineering and a self‐adapted reduction–oxidation reconstruction balance in improving electrocatalytic reactivity and long‐term stability.

## 2 Results and Discussion

### 2.1 Catalysts Screening and Morphological/Structural Characterizations

**FIGURE 1:**
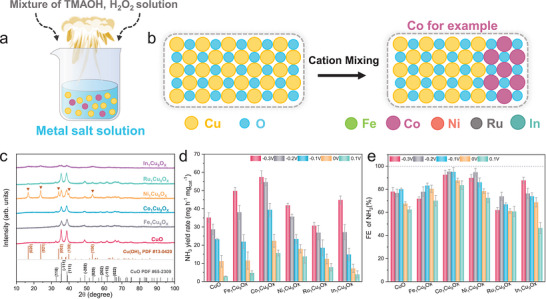
Structural characterizations and NO_3_^−^RR performances of Cu‐based M_1_Cu_9_O_X_ (M is one of Fe, Co, Ni, Ru, In) catalysts. Schematic illustration of (a) synthetic strategy and (b) structure of Cu‐based M_1_Cu_9_O_X_ catalysts; (c) X‐ray diffraction (XRD) patterns of Cu‐based M_1_Cu_9_O_X_ catalysts; (d) NH_3_ yield rate; (e) Faradaic efficiency (FE) of NH_3_ in 1 M KOH+100 mM KNO_3_ after 3600 s electrolysis from 0.1 to −0.3 V vs. RHE, including standard deviations from three independent measurements (all catalysts are pre‐activated in KOH by cyclic voltammetry (CV) cycles sweeping from 0.3 to −0.7 V vs. RHE and in KOH/KNO_3_ electrolyte by chronoamperometry at −0.2 V for 1 h).

Cu‐based oxide catalysts were employed as a representative platform to elucidate the synergistic interactions between copper and secondary metals and the dynamic evolution of structural configurations during electrocatalytic NO_3_^−^RR. All catalysts were prepared through a one‐step, ambient‐condition chemical oxidation of Cu(II) and secondary metal ions in an aqueous solution containing tetramethylammonium (TMA^+^) as a surfactant and dispersant (Figure 1a) [20, 21]. The alkaline medium promoted the formation of negatively charged multi‐metal oxides ((M_x_Oᵧ)^n−^), which subsequently self‐assembled with TMA^+^ cations to yield stable colloidal suspensions within seconds. This rapid, single‐step approach offers excellent compositional flexibility, enabling the incorporation of more than 30 metallic elements. Given the reproducibility and compatibility of this synthesis strategy in accommodating multiple metal species with tunable compositions and spatial distributions within a single framework, Fe, Co, Ni, Ru, and In were selected as secondary metal candidates to probe their synergistic interactions with Cu in electrocatalytic NO_3_^−^RR (Figure 1b). Fe, Co, Ni, and Ru were selected as prototypical d‐block transition metals owing to their partially filled *d* orbitals, which facilitate metal–H bond formation and thereby promote H_2_O dissociation and *H generation [18, 22, 23]. The introduction of Ru favors *H formation by lowering the barrier for H–H coupling. Conversely, as a representative p‐block metal, In enhances the initial adsorption and activation of nitrate, and its intrinsically weak H binding is advantageous for suppressing the competing hydrogen evolution reaction (HER) [24]. From a kinetic perspective, during the practical oxidation synthesis process, differences in oxidation rates and elemental diffusion behavior played an important role in elemental segregation and secondary‐phase formation. X‐ray diffraction (XRD) patterns of all as‐prepared samples exhibited characteristic peaks of CuO (JCPDS Card NO: 65–2309), confirming successful synthesis (Figure 1c). The peaks at 2𝜃 = 16.7, 23.8, 34.1, 39.8 degree, corresponding to Cu(OH)_2_ (JCPDS Card NO: 13–0420), are related to the hydroxide‐stabilizing ability of the second metal. Among all secondary incorporation species, Ni^2+^ possesses the strongest tendency to form stable hydroxide structures, which significantly delays CuO crystallization and leads to the most pronounced Cu(OH)_2_ diffraction signals, whereas Co and Fe promote strong Cu–O–M interactions and facilitate oxide or spinel‐like phase formation [18, 25]. In contrast to Fe, Co, Ni, and Ru modified samples that preserve the bulk CuO crystalline framework, In incorporation significantly suppresses CuO crystallization, leading to reduced long‐range order and weakened XRD diffraction intensity, despite the presence of CuO as the dominant phase.

The electrochemical NO_3_^−^ reduction performance was assessed in an H‐type cell using a conventional three‐electrode configuration under ambient conditions. According to the Pourbaix diagram, Cu‐based oxides or hydroxides are thermodynamically unstable and tend to transform into other phases under alkaline NO_3_^−^RR conditions [26]. Therefore, all catalyst electrodes were pre‐activated in KOH and KOH+KNO_3_ aqueous electrolyte. Ultraviolet‐visible (UV‐vis) spectrophotometry, along with calibration curves, was employed to quantify the concentrations of NO_3_^−^ and possibly generated NH_3_, NO_2_^−^, and N_2_H_4_ (Figures S1–S4). Linear sweep voltammetry (LSV) curves demonstrated that all composites had ammonia‐producing activity and catalytic potential (Figure S5). Among all composites, the Co_1_Cu_9_O_X_ and Ni_1_Cu_9_O_X_ catalysts exhibited superior NO_3_^−^RR performances, with more positive onset potentials, higher NH_3_ yield, and Faradaic efficiency (FE) values (Figure 1d,e). The Fe_1_Cu_9_O_X_ and In_1_Cu_9_O_X_ catalysts presented slightly higher NH_3_ FE and yield at certain applied potentials compared with CuO_X_. Conversely, the Ru_1_Cu_9_O_X_ catalyst exhibited the poorest NO_3_^−^RR performance, with the lowest NH_3_ FE and yield. Excessively strong metal–H bonds hindered ammonia desorption by promoting the competing HER. Among various metals, Co imparted strong synergistic effects with Cu that markedly boosted catalytic performance. Owing to its electronic configuration (3d^7^4s^2^), Co excelled at generating reactive H species via water dissociation, thereby supplying abundant protons for NO_3_^−^ reduction while suppressing competitive HER [16, 27].

**FIGURE 2:**
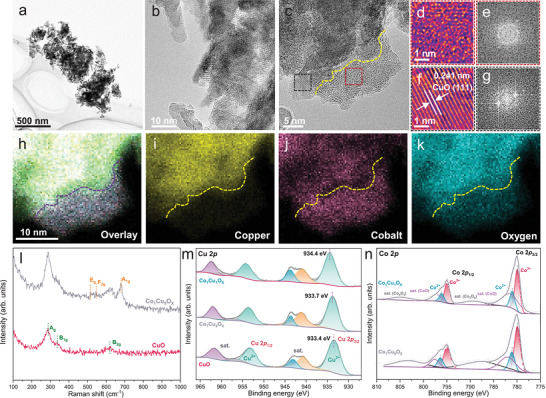
Morphological and structural characterization of Co_1_Cu_9_O_X_. (a, b) TEM images and (c) HR‐TEM images, along with the enlarged region of (d) red and (f) black areas; Corresponding fast Fourier transform (FFT) patterns acquired from different marked regions; (h–k) Corresponding energy dispersive spectroscopy (EDS) element mappings of Co_1_Cu_9_O_X_; (l) Raman spectra of CuO and Co_1_Cu_9_O_X_; (m, n) XPS spectra of Cu 2*p* and Co 2*p* in CuO, Co_1_Cu_9_O_X,_ and Co_1_Cu_1_O_X_.

To clarify the origin of the observed performance difference, the morphology and phase composition of CuO and all M_1_Cu_9_O_X_ catalysts were characterized by scanning transmission electron microscopy (STEM), HR‐TEM, EDS mapping analyses, and XPS analysis (Figure 2 and Figures S6–S11). As revealed by morphology analyses, CuO and all M_1_Cu_9_O_X_ catalysts featured a similar nanosheet‐like nanocrystalline morphology, with no significant differences in particle size or overall architecture. Therefore, morphology/particle size‐related effects can be reasonably excluded as the primary origin of the activity differences. Elemental mapping further unveils the elemental distribution throughout the M_1_Cu_9_O_X_ matrix. Compared to Fe_1_Cu_9_O_X_, Ru_1_Cu_9_O_X_, and In_1_Cu_9_O_X,_ with relatively uniform distribution of elements, Ni_1_Cu_9_O_X_ presented a heterogeneous structure similar to that of Co_1_Cu_9_O_X_, with distinct NiO_X_/CoO_X_ domains embedded within the CuO matrix due to thermodynamic factors and unequal feeding ratios. The generation of interfacial heterostructures can provide additional active sites and facilitate interfacial charge transfer, which may be one of the reasons for its high activity of NO_3_^−^RR performance on Ni_1_Cu_9_O_X_ and Co_1_Cu_9_O_X_ [28, 29]. XPS analysis provided the differences in surface chemical states among these catalysts. Cu and the incorporated Fe, Co, Ni, Ru, and In species all featured oxidized states, corroborating the successful incorporation of the secondary metals into the Cu‐based oxide catalysts [30]. In particular, the O 1*s* spectra of Ni_1_Cu_9_O_X_ and In_1_Cu_9_O_X_ displayed significantly higher proportions of surface hydroxyl (‐OH) species than the other samples [31, 32]. This observation was in excellent agreement with the Cu(OH)_2_ diffraction peaks identified by XRD, confirming the stronger hydroxide‐stabilizing capability of Ni and In‐related composites. While surface hydroxyl groups can promote water activation, excessive hydroxyl stabilization may impede the formation of the active phase and alter the local reaction environment [11]. Importantly, despite the comparable heterostructure of Ni_1_Cu_9_O_X_ and its higher surface hydroxyl content, Co_1_Cu_9_O_X_ still exhibited superior NO_3_^−^RR performance. These results suggest that the enhanced catalytic activity does not arise solely from the heterostructure or hydroxyl content, thereby motivating a more comprehensive investigation into the unique structural and heterostructure characteristics of Co_1_Cu_9_O_X_.

For Co_1_Cu_9_O_X_, a nanocrystalline structure was observed on a large scale, as presented in Figure 2a,b. In high‐resolution observations, a crystalline‐amorphous heterogeneous matrix and uneven elemental distribution were observed, as shown in Figure 2c,d–k, demonstrating that Co_1_Cu_9_O_X_ consisted of heterogeneous contacts rather than a fully uniform solid‐solution structure at the nanoscale. The CoO_X_ species exhibited blurred diffraction rings and irregular lattice contrasts, as shown in Figure 2d,e, indicating an amorphous structure with low crystallinity. Occasional short‐range ordered domains were observed, whereas the overall phase remained predominantly amorphous with partial nanocrystalline features. Well‐defined CuO (111) facets were identified in the crystalline domains, whereas the edge regions exhibited an amorphous character containing only Co and O. This observation was further corroborated by powder XRD and TEM observation (Figures S12 and S13), revealing characteristic diffraction peaks of CuO, while no discernible reflections associated with CoO_X_ were detected [33]. The diffraction peaks corresponding to the (110), (‐111), (111), (‐202), (020), (202), and (‐113) planes belonged to monoclinic CuO (JCPDS Card NO: 65–2309). The intensity and sharpness of the diffraction peaks decreased significantly with increasing Co content, indicating the introduction of a higher proportion of amorphous components. Additionally, Co_1_Cu_1_O_X_ exhibited homogeneous elemental distribution without apparent phase separation (Figure S14), originating from comparable precursor concentrations that favor synchronous nucleation and growth of Co and Cu species. Conversely, under Cu‐rich conditions (Co_1_Cu_9_O_X_), the limited solubility of Co in the CuO lattice, along with dominant Cu─O─Cu interactions during nucleation, drove phase segregation and led to the formation of a CuO/CoO_X_ composite with an inhomogeneous elemental distribution.

The Raman spectra featured prominent peaks centered at 288, 337, and 624 cm^−1^, corresponding to the A*_g_*, B_1_ *_g_*_,_ and B_2_ *_g_* modes of monoclinic CuO, respectively, for both CuO and Co_1_Cu_9_O_X_ catalysts [34, 35]. As expected, the additional peaks at 515, 546, and the dominant 679 cm^−1^, matching the E*_g_*, F_2_ *_g_*, and A_1_ *_g_* modes of CoO, were observed for the Co_1_Cu_9_O_X_ catalyst, corroborating the formation of heterogeneous structures (Figure 2l) [36]. Significant signals from target elements, including Cu 2*p*, O 1*s*, and Co 2*p* (Figure S15a), were observed in the XPS survey spectra. The Cu 2*p* spectra revealed obvious characteristic satellite peaks, indicating that copper mainly existed in the Cu^2+^ oxidation state (Figure 2m) [34, 37]. Auger electron spectroscopy (AES) (Figure S15b) further demonstrated the predominant presence of Cu^2+^ in the original catalysts. The Co 2*p* spectra (Figure 2n) exhibited mixed paired Co^2+^/Co^3+^ peaks [38, 39]. The O 1*s* spectrum revealed three characteristic peaks at 533.35 eV (adsorption oxygen), 531.65 eV (surface −OH), and 529.75 eV (lattice oxygen) (Figure S15c) [31, 32]. The decreased and increased electron density on Cu and Co, respectively, suggested electron transfer from Cu atoms to Co atomic sites, indicating a strong interaction between Cu and Co species in the composites.

### 2.2 Identification of Adaptive Dynamic Evolution During NO_3_^−^RR

**FIGURE 3:**
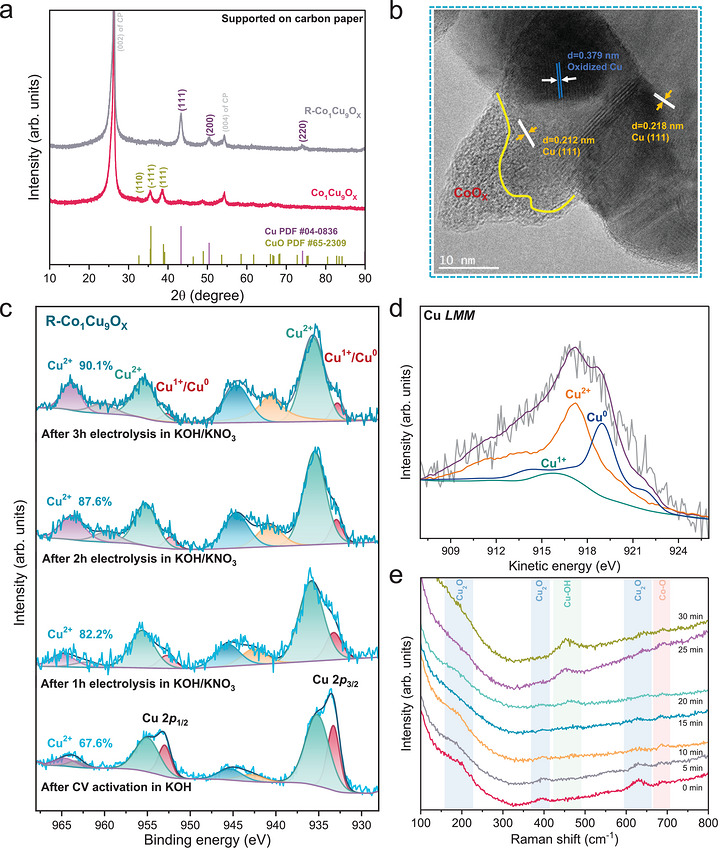
Morphological and structural characterization of reconstructed Co_1_Cu_9_O_X_. (a) XRD pattern of Co_1_Cu_9_O_X_ and R‐Co_1_Cu_9_O_X_ (Co_1_Cu_9_O_X_ after pre‐activation: 50 cycles of CV activation from 0.3 V to −0.7 V vs. RHE in 1 M KOH and 1 h of chronoamperometric electrolysis in 1.0 M KOH+100 mM KNO_3_); (b) HR‐TEM image of R‐Co_1_Cu_9_O_X_; (c) Quasi operando XPS analyses of Cu 2*p* in Co_1_Cu_9_O_X_ only after CV activation in KOH and after both CV activation in KOH and electrolysis in KOH/KNO_3_ electrolyte with different durations for 1, 2, and 3 h; (d) Cu LMM AES XPS spectra with NLS‐based curve fitting of R‐Co_1_Cu_9_O_X_; (e) In‐situ Raman spectra of Co_1_Cu_9_O_X_ under –0.2 V vs. RHE with incremental operation time in 1.0 M KOH+100 mM KNO_3_.

Cu exhibits multiple valence states because the energies of its 3*d* and 4*s* orbitals are relatively close, enabling flexible coordination and adsorption with reactants [40]. However, under electrochemical conditions, Cu‐based catalysts often undergo self‐reconstruction during activation and reaction, making it difficult to precisely identify the true active sites, particularly in high‐valence copper oxides. While Pourbaix diagrams provide insight into the thermodynamically stable oxidation states and phases at different potentials and pH values, they do not account for the kinetics of redox transitions or the resulting structural dynamics of the catalyst. Unless otherwise specified, the reconstructed catalysts after pre‐activation, denoted as R‐, were obtained after CV activation in KOH followed by 1 h NO_3_^−^RR electrolysis in KOH/KNO_3_, such as R‐CuO_X_ and R‐Co_1_Cu_9_O_X_. As seen from the XRD patterns of CuO and Co_1_Cu_9_O_X_ after pre‐activation reconstruction, the diffraction peak of CuO at 2𝜃 = 35.4°(‐111) and 38.6° (111) almost disappeared, while distinct diffraction peaks at 43.4° (111), 50.4° (200) and 74.2° (220) corresponding to metallic Cu were detected, suggesting that reductive reconstruction generated metallic Cu phases in the bulk region during the NO_3_^−^RR process (Figure 3a and Figure S16) [2, 34]. Further and visualized surface structural evidence was provided by local HR‐TEM analysis of R‐Co_1_Cu_9_O_X_. Besides lattice fringes corresponding to metallic Cu (111), additional fringes with larger interplanar spacings of 0.379 nm were observed, which may be associated with local oxidized or hydroxylated Cu–O coordination (Figure 3b) [11].

Interestingly, although bulk‐sensitive XRD identified the formation of crystalline metallic Cu domains, surface‐sensitive quasi‐in situ XPS, complemented by local TEM and operando Raman analyses, revealed a spatially heterogeneous reconstruction behavior involving a Cu–OH‐rich oxidized Cu surface. After electrochemical activation by cyclic voltammetry in KOH, the Cu 2*p* spectra exhibited two dominant pairs of peaks centered at approximately 933.5/953.0 eV and 935.5/955.0 eV, assigned to Cu^1+^/Cu^0^ and Cu^2+^, respectively (Figures 3c and S17a). Both catalysts initially underwent reductive reconstruction in KOH electrolyte, while Co_1_Cu_9_O_X_ presented a substantially higher surface Cu^0^/Cu^+^ fraction than CuO. Unexpectedly, subsequent NO_3_^−^ electroreduction in KOH/KNO_3_ did not further enrich surface metallic Cu as generally expected, despite the highly reductive operating conditions. TEM measurements and EDS element maps revealed differences in morphology and oxygen content between post‐activation in KOH and electrolysis for NO_3_^−^RR (Figures S17b–i, S18 and S19). To be specific, after CV activation in KOH, the composites evolved into highly crystalline metallic Cu nanodomains with reduced oxygen content, indicating reductive reconstruction. In contrast, after NO_3_^−^RR electrolysis at −0.2 V vs. RHE in KOH/KNO_3_, re‐oxidation of the surface was observed, which is likely associated with NO_3_^−^RR‐induced surface restructuring together with the re‐oxidation of newly exposed surface sites [1, 34]. Quantitative XPS fitting indicated that the Cu^2+^ content of CuO increased from 84.2% after activation in KOH to 90.2% after 3 h NO_3_^−^ electroreduction, while Co_1_Cu_9_O_X_ showed a more pronounced evolution from 67.6% to 90.1% (Figures 3c, S17a). Cu LMM AES analysis with non‐linear least‐squares (NLS) fitting, together with the pronounced Cu 2*p* satellite features observed after pre‐activation, further confirmed the dominant surface presence of Cu^2+^ species on both R‐CuO_X_ and R‐Co_1_Cu_9_O_X_ (Figure S20, Figure 3d). Consistently, the positively shifted Cu 2*p* binding energies relative to original CuO suggested a more electron‐deficient Cu environment characteristic of hydroxyl‐coordinated Cu species, while the enhanced hydroxyl‐related O 1*s* signal revealed pronounced surface ‐OH enrichment in both R‐CuO_X_ and R‐Co_1_Cu_9_O_X_ (Figures S15c, S21) [31, 32].

Since metallic Cu is Raman‐inactive, in situ electrochemical Raman spectroscopy provides a more accessible way to capture the dynamic evolution of oxidized or hydroxylated Cu species rather than metallic Cu itself (Figure 3e). After cyclic voltammetry (CV) activation in KOH, three Raman bands centered at approximately 190, 390, and 630 cm^−1^ were detected, which can be assigned to Cu_2_O‐related vibrational modes [34, 41]. With increasing reaction time under NO_3_^−^RR conditions, these Cu_2_O‐related bands gradually weakened and eventually became indistinct, indicating continuous surface reconstruction. Meanwhile, a new weak band appeared at around 455 cm^−^^1^, which can be attributed to Cu–O–H‐related vibrations, suggesting the formation of surface Cu–OH species [1, 42]. Taken together, these results demonstrated that the reconstructed surface dynamically evolved into a surface‐localized Cu–OH‐rich oxidized Cu state during NO_3_^−^RR. The NH_3_ yield rates and FEs on reconstructed CuO and Co_1_Cu_9_O_X_ at different NO_3_^−^ electroreduction durations were also evaluated. The similar performance results ruled out a negative impact of Cu surface reconstruction during electrolysis on catalytic performance (Figure S22).

It's worth noticing that Co incorporation as CoO_X_ seemed to markedly accelerate this reconstruction process, as presented in the more pronounced evolution of surface Cu^2+^. The higher initial low‐valence Cu content generated in R‐Co_1_Cu_9_O_X_ heterostructure during alkaline activation in KOH electrolyte provided a more reconstruction‐responsive surface, while the electronic interaction between Co and Cu further facilitated surface re‐oxidation/hydroxylation under reaction conditions. Consequently, Co_1_Cu_9_O_X_ heterostructure underwent a more rapid evolution toward the catalytically relevant hydroxyl‐rich oxidized Cu^2+^ interface under a more strongly perturbed interfacial microenvironment during NO_3_^−^ electroreduction for NH_3_ generation. Ni_1_Cu_9_O_X_, which possesses a similar heterostructure configuration but exhibits more pronounced diffraction features associated with hydroxylated Cu‐related structures, showed substantially inferior NO_3_^−^RR performance. This observation underscores that the catalytic enhancement is primarily governed by the in‐situ dynamic reconstruction process, rather than by the mere presence of pre‐existing hydroxylated Cu species. The reconstructed surface‐localized Cu–OH‐rich oxidized interfacial environment formed under reaction conditions likely plays an important role in promoting nitrate electroreduction.

Collectively, these results suggested a self‐adaptively dynamic reconstruction mechanism with a continuous balance among NO_3_^−^ electroreduction, in which the initially generated metallic Cu species did not remain the dominant surface state under operating conditions but self‐adaptively evolved into a surface‐localized Cu–OH‐rich oxidized Cu state in the NO_3_^−^ containing electrolyte. During NO_3_^−^RR, although NO_3_^−^ is reduced overall, nitrate and its partially reduced intermediates (such as NO_2_^−^, NO, and adsorbed *NO species) are strong oxygen‐containing oxidants [43]. Their interaction with the copper surface can locally withdraw electron density, resulting in partial oxidation of Cu^0^ or Cu^+^ back to higher oxidation states. In addition, continuous NH_3_ generation locally increased the OH^−^ concentration, thereby promoting the hydroxylation of surface Cu species through reactions between OH^−^ and Cu_2_O/Cu^2+^, ultimately driving the self‐adaptive formation of a surface‐localized hydroxyl‐rich oxidized Cu steady state [1, 44, 45]. Notably, Co incorporation as CoO_X_ significantly accelerates this interfacial reconstruction process through strong electronic coupling and more intense interfacial microenvironment change during NO_3_^−^ electroreduction for NH_3_ generation. Such self‐adaptive, surface Cu–OH‐rich oxidized Cu species and intrinsic metallic Cu reconstruction from the Co_1_Cu_9_O_X_ heterostructure (as in CuO/CoO_X_) were expected to optimize the adsorption of reaction intermediates and facilitate their hydrogenation through a dynamically regulated interfacial catalytic environment, thereby being responsible for the observed high nitrate‐to‐ammonia conversion rate [9, 46].

### 2.3 Evaluation of Electrocatalytic NO_3_^−^ RR Performance

**FIGURE 4:**
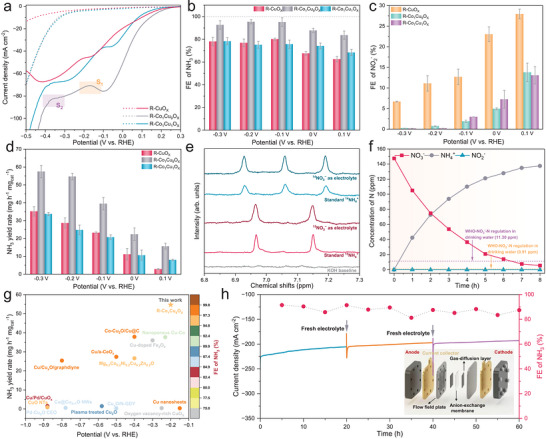
Electrocatalytic NO_3_^−^RR performance of R‐Co_1_Cu_9_O_X_ catalysts. (a) LSV curves at a scan rate of 10 mV s^−1^ in 1 M KOH solution without (dotted line) and with (solid line) 100 mM of NO_3_^−^; (b–d) Faradaic efficiency (FE) of NH_3_, FE of NO_2_^−^, and NH_3_ yield rate, after 3600 s of electrolysis from 0.1 to −0.3 V vs. RHE of R‐CuO_X_, R‐Co_1_Cu_9_O_X_, and R‐Co_1_Cu_1_O_X_ (error bars (mean ± standard deviation) are obtained based on three independent electro‐catalytic experiments); (e) ^1^H NMR spectra of standard ^15^NH_4_^+^ and ^14^NH_4_^+^ and generated ^15^NH_4_^+^ and ^14^NH_4_^+^ with ^15^NO_3_^–^ and ^14^NO_3_^−^ as the nitrogen sources, R‐Co_1_Cu_9_O_X_ as catalysts; (f) Time‐resolved concentrations of NO_3_^−^, NO_2_^−^, and NH_4_^+^ on R‐Co_1_Cu_9_O_X_ (2*1.5 cm^−2^) at −0.2 V vs. RHE; (g) Performance comparison of the optimized R‐Co_1_Cu_9_O_X_ with previously reported Cu‐based electrocatalysts for NO_3_^−^RR; (h) Long‐term stability assessment and the time‐resolved NH_3_ FE of the R‐Co_1_Cu_9_O_X_ catalyst in the customized flow cell with a three‐electrode design by Suzhou Sinero Technology Co., Ltd (China) (inset: schematic diagram of anion exchange membrane flow cell and flow field structure).

To investigate the influence of Co content and the formation of heterostructure in Co_1_Cu_9_O_X_ on NO_3_^−^ RR performance, a series of Cu‐based composite catalysts with varying Co proportions was synthesized by adjusting the Co/Cu precursor feeding ratios while maintaining all other conditions constant, as mentioned before (Figure S12). As shown in Figure S23, when both yield and FE were considered, optimal performance was achieved at a Co/Cu metal salt precursor molar feeding ratio of 1:9. Conversely, excessive Co content (50 at.%) resulted in uniform elemental distribution without apparent phase separation but deteriorated NO_3_^−^RR activity due to enhanced competitive hydrogen evolution. Figure 4a presents LSV curves of R‐CuO_X_, R‐Co_1_Cu_9_O_X_, and R‐Co_1_Cu_1_O_X_ recorded in 1.0 M KOH with and without 100 mM NO_3_^−^. Upon introduction of NO_3_^−^, all catalysts recorded markedly enhanced current densities and distinct cathodic features relative to those measured in NO_3_^−^‐free electrolyte, generally indicating the onset of early‐stage NO_X_ activation and subsequent NO_3_^−^ reduction [6]. Notably, the R‐Co_1_Cu_9_O_X_ exhibited a more pronounced S1 peak at ‐0.17 V, whereas only a weak anodic feature appeared near ‐0.11 V during the reverse scan of CV (Figure S24). Given the multi‐electron and highly irreversible nature of NO_3_^−^RR, this voltammetric response can unlikely be attributed to discrete redox couples associated with NO_3_^−^/NO_2_^−^ or NO_2_^−^/NH_3_ transformations, which typically manifest as broad catalytic waves rather than well‐defined peaks. Therefore, the cathodic feature near ‐0.17 V was more reasonably assigned to the nitrate‐induced adaptive surface electrochemical reaction, enabled by strong NO_3_^−^ adsorption and stabilization of reduced Cu species. As a whole, these observations suggest that the CV features primarily reflect nitrate‐triggered adaptive reconstruction of the Cu surface coupled with early‐stage NO_X_ activation, which establishes the catalytically active state for subsequent ammonia formation.

Chronoamperometric tests were performed at various potentials for 1 h in 1 M KOH containing 100 mM NO_3_^–^ after electrochemical pre‐activation to quantify NO_3_^−^RR activity (Figure S25). Figure 4b–d summarizes the FEs of NH_3_ and NO_2_^−^, along with the NH_3_ yield rates, for R‐CuO_X_, R‐Co_1_Cu_9_O_X_, and R‐Co_1_Cu_1_O_X_ catalysts at different applied potentials. The highest FEs of NO_2_^−^ were observed for R‐CuO_X_ over the examined potential range, indicating efficient NO_3_^−^‐to‐NO_2_^−^ conversion but limited activity toward subsequent NO_2_^−^ reduction to NH_3_ at low overpotentials. Upon incorporation of Co, the NH_3_ FEs for both R‐Co_1_Cu_9_O_X_ and R‐Co_1_Cu_1_O_X_ increased markedly, suggesting effective suppression of NO_2_^−^ accumulation. Notably, R‐Co_1_Cu_9_O_X_ achieved a maximum NH_3_ FE of 95.4% at −0.2 V vs. RHE, substantially exceeding those of R‐CuO_X_ (76.8%) and R‐Co_1_Cu_1_O_X_ (75.2%) (Figure 4b). Consistently, R‐Co_1_Cu_9_O_X_ heterostructure exhibited an NH_3_ yield rate of 54.68 mg h^−1^ mg_cat._^−1^ at −0.2 V vs. RHE, which was approximately 1.90 and 2.20 times higher than those of R‐CuO_X_ (28.72 mg h^−1^ mg_cat._^−1^) and R‐Co_1_Cu_1_O_X_ (24.85 mg h^−1^ mg_cat._^−1^), respectively (Figure 4d). Accordingly, NO_3_^−^RR on R‐Co_1_Cu_9_O_X_ followed a synergistic catalytic pathway, where Cu sites preferentially facilitated the reduction of NO_3_^−^ to NO_2_^−^, while the incorporation of CoO_X_ in R‐Co_1_Cu_9_O_X_ promoted the subsequent conversion of NO_2_^−^ to NH_3_. Moreover, no N_2_, H_2_, or N_2_H_4_ was detected during electrocatalysis over R‐Co_1_Cu_9_O_X_ at −0.2 V vs. RHE (Figure S26). Furthermore, CoO_X_ alone was inactive in this system, and the influence of the carbon paper (CP) substrate on catalytic performance was excluded (Figure S27).

To confirm the nitrogen origin of NH_3_, isotope labeling experiments were performed using K^14^NO_3_ and K^15^NO_3_ as nitrogen sources (Figure 4e). The generated ^14^NH_4_^+^/^15^NH_4_^+^ signals from the electrolysis of ^14^NO_3_^−^/^15^NO_3_^−^ presented the characteristic triplet and doublet peaks, respectively, consistent with those of standard ^14^NH_4_^+^/^15^NH_4_^+^, revealing that all detected NH_3_ originated exclusively from nitrate rather than external contamination. Consistently, a control experiment conducted in KOH without NO_3_^−^ revealed no detectable NH_3_ (Figure 4e). Meanwhile, the concentration of NH_4_^+^ was determined by ^1^H nuclear magnetic resonance (NMR, 399.78 MHz) spectroscopy to validate the accuracy of the colorimetric method (Figure S28a,b) [28]. The quantification results for both ^14^NH_4_^+^ and ^15^NH_4_^+^ closely matched those obtained from UV‐vis analysis, indicating the reliability of NH_3_ detection and quantitation in this study (Figure S28c). Figure 4g and Table S1 benchmark the nitrate reduction performance of R‐Co_1_Cu_9_O_X_, in terms of NH_3_ yield and FE, against previously reported catalysts. R‐Co_1_Cu_9_O_X_ exhibited superior performance at a lower potential than other Cu‐based catalysts.

Continuous recycling tests were conducted to evaluate the electrochemical durability of the R‐Co_1_Cu_9_O_X_ in an H‐type reactor (Figure S29a). After five cycles of operation, no distinct attenuation was observed in the yield rate and FE of NH_3_ (Figure S29b). To further assess the practical viability of R‐Co_1_Cu_9_O_X_ for ammonia electrosynthesis, its performance was evaluated in a flow‐cell configuration. Relative to the conventional H‐cell, the flow‐cell setup delivered substantially higher current densities, as evidenced by both linear sweep voltammetry and chronoamperometric measurements (Figure S30 and Figure 4h). Notably, under continuous operation at −0.2 V vs. RHE in a customized three‐electrode flow cell, R‐Co_1_Cu_9_O_X_ exhibited sustained catalytic performance in 1.0 M KOH containing 100 mM KNO_3_. The catalyst maintained a high current density and FE over 60 h of electrolysis without discernible degradation (Figure 4h), underscoring its sustained operational stability and promise for practical nitrate‐to‐ammonia conversion. After stability measurements, no significant changes in the crystal structure and surface chemical composition were observed, as shown in Figure S31, still featuring bulk metallic Cu^0^ and surface hydroxylated oxidized Cu species. Furthermore, the morphological structure and heterojunction characteristics were well maintained (Figure S32). The LSV and chronoamperometry curves of R‐Co_1_Cu_9_O_X_ in 1.0 M KOH containing different concentrations of NO_3_^−^ are shown in Figure S33a,b. Given the wide variation in NO_3_^−^ concentrations across different nitrate sources, the dependence of R‐Co_1_Cu_9_O_X_ NO_3_^−^RR performance on nitrate concentrations was systematically examined. As the concentration increased, the current density of electrocatalysis increased. The NO_3_^−^RR activity accelerated with increasing NO_3_^−^ concentration in the range of 5–200 mM NO_3_^−^, as evidenced by the higher current density and positive onset potential. The value of NH_3_ FE increased from 55.50% (5 mM NO_3_^−^) to 95.40% (100 mM NO_3_^−^), and then slightly decreased to 94.87% (200 mM NO_3_^−^). For low NO_3_^−^ concentrations, R‐Co_1_Cu_9_O_X_ exhibited a lower NH_3_ FE due to enhanced hydrogen evolution (Figure S33c). The slight decrease in NH_3_ FE at elevated NO_3_^−^ concentrations can be attributed to the partial deactivation of active sites, caused by the delayed desorption of generated NH_3_ from the catalyst surface. Collectively, R‐Co_1_Cu_9_O_X_ maintained a high NH_3_ FE in 100 mM NO_3_^−^ compared with other measured concentrations. Therefore, 1 M KOH containing 100 mM KNO_3_ was selected as the optimized electrolyte conditions to achieve the highest performance of the R‐Co_1_Cu_9_O_X_ catalyst. Given that NO_3_^−^‐N concentrations in various waste streams, including textile and heavy‐industry wastewater, typically range from 10 to 100 mM, R‐Co_1_Cu_9_O_X_ exhibits promising potential for practical NO_3_^−^‐containing wastewater treatment [47]. The nitrate removal ability of R‐Co_1_Cu_9_O_X_ catalyst was investigated in 140 ppm NO_3_^−^‐N (10 mM NO_3_^−^). After 7 h, the levels of NO_3_^−^‐N (7.45 ppm) and NO_2_^−^‐N (0.003 ppm) decreased to values below the threshold standard determined by the World Health Organization guideline for drinking water (Figure 4f), demonstrating the excellent NO_3_^−^ removal performance and promising application potential of R‐Co_1_Cu_9_O_X_ for treating NO_3_^−^ ‐containing wastewater.

### 2.4 Mechanistic Insights

**FIGURE 5:**
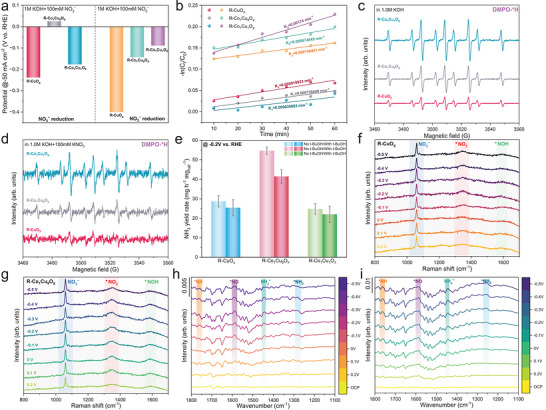
Mechanism investigation and kinetic study. (a) Potentials extracted from LSV curves at a current density of ‐50 mA cm^−2^ and (b) the reaction constants K (K_1_ for NO_3_^−^‐to‐NO_2_^−^ conversion and K_2_ for NO_2_^−^‐to‐NH_3_ conversion on R‐CuO_X_, R‐Co_1_Cu_9_O_X_, and R‐Co_1_Cu_1_O_X_; Operando EPR spectra of R‐CuO_X_, R‐Co_1_Cu_9_O_X_, R‐Co_1_Cu_1_O_X_ after electrocatalysis for 10 min at −0.2 V vs. RHE in 1.0 M KOH (c) without and (d) with 100 mM KNO_3_; (e) the NH_3_ yield rate of R‐CuO_X_, R‐Co_1_Cu_9_O_X_, and R‐Co_1_Cu_1_O_X_ at −0.2 V vs. RHE with and without 0.5 M t‐BuOH in 1.0 M KOH + 100 mM KNO_3_; In‐situ Raman spectra of (f) R‐CuO_X_ and (g) R‐Co_1_Cu_9_O_X_ at different potentials in 1 M KOH + 100 mM NO_3_^−^ solution; In situ FTIR spectra of (h) R‐CuO_X_ and (i) R‐Co_1_Cu_9_O_X_ at different potentials in 1 M KOH + 100 mM NO_3_^−^ solution.

To elucidate the origin of improved activity on R‐Co_1_Cu_9_O_X_, the electrochemical double‐layer capacitance (C_dl_) was determined from CV recorded in the non‐faradaic region at varying scan rates (Figure S34). The capacitance was then used as an estimate of the electrochemically active surface area (ECSA). R‐Co_1_Cu_9_O_X_ (0.144 mF cm^−2^) and R‐Co_1_Cu_1_O_X_ (0.102 mF cm^−2^) presented larger C_dl_ than R‐CuO_X_ (0.098 mF cm^−2^), as shown in Figure S35a, suggesting that Co incorporation and heterointerface formation increased the amount of effective active sites. After normalization to ECSA, R‐Co_1_Cu_9_O_X_ exhibited the highest NH_3_ yield rate (Figure S35b), demonstrating its superior intrinsic NO_3_^−^ RR activity. Furthermore, electrochemical impedance spectroscopy measurements revealed that R‐Co_1_Cu_9_O_X_ delivered the lowest charge transfer resistance (R_ct_) (Figure S36), suggesting more efficient interfacial charge‐transfer kinetics during NO_3_^−^ reduction. A comparison of the LSV curve (Figure S37) derived overpotentials at −50 mA cm^−2^ for different catalysts in NO_3_^−^ and NO_2_^−^ solutions is shown in Figure 5a. Among all catalysts, R‐Co_1_Cu_9_O_X_ exhibited the most positive potential (25.0 mV) in NO_3_^−^ solution, while R‐Co_1_Cu_1_O_X_ presented the most positive potential (−88.9 mV) in NO_2_^−^ solution, indicating that R‐Co_1_Cu_9_O_X_ and R‐Co_1_Cu_1_O_X_ exhibited the lowest energy barrier for NO_3_^−^ and NO_2_^−^ reduction, respectively [48]. Therefore, R‐Co_1_Cu_9_O_X_ achieved a balance between NO_3_^−^ and NO_2_^−^ reduction, thus obtaining the optimal NH_3_ generation activity. To elucidate the NO_3_^−^RR pathway, the reaction rate constants (K) for NO_3_^−^‐to‐NO_2_^−^ (K_1_) and NO_2_^−^‐to‐NH_3_ (K_2_) were determined. As shown in Figure 5b, CuO_X_ indicated the highest K_1_ with a K_1_/K_2_ ratio of 1.23, suggesting that R‐CuO_X_ facilitated the NO_3_^−^‐to‐NO_2_^−^ conversion. R‐Co_1_Cu_1_O_X_ had the highest K_2_ value with a K_1_/K_2_ ratio of 0.36, indicating its high activity for NO_2_^−^‐to NH_3_ conversion. Notably, the K_1_/K_2_ value of R‐Co_1_Cu_9_O_X_ (0.76) was less than that of R‐CuO_X_ (1.67) but greater than that of R‐Co_1_Cu_1_O_X_ (0.36), indicating the promotion of NO_3_^−^RR by Co species cooperation and heterointerface through simultaneous acceleration of NO_3_^−^‐to‐NO_2_^−^ and NO_2_^−^‐to‐NH_3_ conversions.

To directly verify the formation and participation of hydrogen radicals during catalysis, spin‐trapping EPR measurements were performed using 5,5‐dimethyl‐1‐pyrroline‐N‐oxide (DMPO) as the trapping agent [23]. Under nitrate‐free conditions, all reconstructed catalysts exhibited characteristic DMPO–*H signals, confirming the generation of hydrogen radicals during electrolysis [49]. The signal intensity followed the order of R‐Co_1_Cu_1_O_X_ > R‐Co_1_Cu_9_O_X_ > R‐CuO_X_, indicating that Co incorporation promoted water dissociation and enhanced *H generation (Figure 5c). Upon introduction of NO_3_^−^, the DMPO–*H signals markedly decreased for all catalysts because of rapid hydrogen consumption during nitrate hydrogenation (Figure 5d). Notably, although R‐Co_1_Cu_1_O_X_ generated the highest amount of hydrogen radicals, R‐Co_1_Cu_9_O_X_ exhibited the most efficient coupling between hydrogen supply and nitrate conversion. This behavior suggested that appropriate Co incorporation not only accelerated H_2_O dissociation but also facilitated interfacial *H rapid spillover, thereby promoting the hydrogenation of nitrogen‐containing intermediates to generate NH_3_. In contrast, excessive Co loading disrupted the balance between hydrogen generation and nitrate activation, leading to inferior NH_3_ synthesis performance. The critical role of active hydrogen species was further confirmed by *H quenching experiments using tert‐butanol (t‐BuOH) as a selective *H scavenger [50]. As shown in Figure S38a,b, the introduction of t‐BuOH led to a pronounced decrease in the current density, indicating that hydrogen‐related surface reactions were significantly suppressed. Correspondingly, both the NH_3_ yield rate and Faradaic efficiency declined markedly after the addition of t‐BuOH (Figure 5e and Figure S38c). Notably, this inhibitory effect was most significant for the R‐Co_1_Cu_9_O_X_ heterostructure, highlighting the essential role of active hydrogen species in its enhanced nitrate reduction performance. These findings are in excellent agreement with the operando EPR results, which directly reveal that an appropriate incorporation of Co and the formation of heterostructure promote the generation of reactive *H species and facilitate its interfacial spillover and efficient utilization. In contrast, excessive Co loading will disrupt the balance between deoxygenation and hydrogenation steps, ultimately leading to diminished ammonia production. This result underscores the importance of precisely tuning the Cu/Co interfacial structure to achieve optimal NO_3_^−^RR performance.

To further probe the evolution of reaction intermediates and *H availability during NO_3_^−^ electroreduction, in‐situ Raman spectra were collected at various potentials (Figure 5f,g) [51]. The prominent band at 1060 cm^−1^ was assigned to the symmetric stretching vibration of NO_3_^−^. With increasing applied potential, new Raman features at 1345 and 1590 cm^−1^ corresponding to *NO_2_ and N═O scaling vibration of *NOH, gradually appeared and intensified [52, 53, 54]. Compared with pristine R‐CuO_X_, the heterostructure exhibited stronger *NO_2_ signals, reflecting an increased surface coverage of NO_2_‐derived intermediates induced by interfacial electronic modulation. Simultaneously, the pronounced features of *NOH in R‐Co_1_Cu_9_O_X_ suggested that subsequent hydrogenation steps were more favorable, allowing deeper reduction toward NH_3_. Rather than desorbing as NO_2_^−^ into the electrolyte, *NO_2_ remained bound on the catalyst surface and underwent further hydrogenation, highlighting the role of the heterojunction in steering selectivity toward deep reduction. This behavior can be attributed to the synergistic cascade effects, including charge transfer, local charge density redistribution, or the presence of bifunctional active sites that simultaneously stabilize nitrogenous intermediates and lower the activation barriers for hydrogenation [5, 37, 54]. In situ ATR‐SEIRAS spectroscopy was performed to further investigate the intermediates at different potentials during the NO_3_^−^RR (Figure 5h,i). Characteristic peaks and bands attributed to the deoxidation intermediates (*NO at 1590 cm^−1^) and hydrogenation intermediates (*NH_2_ at 1270 cm^−1^, *NH at 1760 cm^−1^, and NH_4_^+^ at 1445 cm^−1^) appeared and gradually increased with the elevated applied potential, indicating that R‐CuO_X_ and R‐Co_1_Cu_9_O_X_ effectively activated NO_3_^−^ to generate hydrogenation intermediates [55, 56, 57, 58, 59]. The emergence of the characteristic band associated with the N–O antisymmetric stretching vibration of *NO indicated the consumption of NO_3_^−^ and its reduction to NO_2_^−^, followed by further deoxygenation steps. Furthermore, the signal intensities of hydrogenation intermediates, including out‐of‐plane *NH_2_ rocking, *NH bending, along with NH_4_^+^ N‐H bending vibrations in R‐Co_1_Cu_9_O_X_, were notably more intense and distinct than those in R‐CuO_X_, verifying the enhanced deoxygenation and hydrogenation ability during the formation of NH_3_ in R‐Co_1_Cu_9_O_X_ [60]. Taken together, the operando EPR, *H quenching, in‐situ Raman, and ATR‐SEIRAS results reveal that the self‐adaptive R‐Co_1_Cu_9_O_X_ heterostructure with mainly Cu/CoO_X_ heterostructure and surface‐localized hydroxylated oxidized Cu species stabilizes nitrogen‐containing intermediates, promotes interfacial *H spillover, and rapid hydrogenation of nitrate‐derived intermediates. The synergistic coupling between deoxygenation and hydrogenation processes in R‐Co_1_Cu_9_O_X_ enables accelerated reaction kinetics and enhanced NH_3_ selectivity during NO_3_^−^RR.

### 2.5 Reaction Pathway Investigation and Theoretical Insights Into NO_3_^−^RR

**FIGURE 6:**
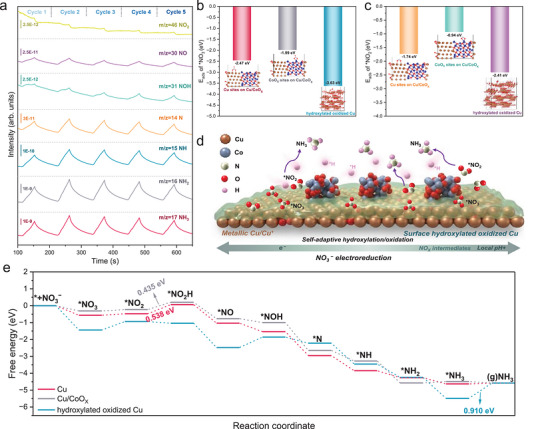
Reaction pathway analysis and schematic illustration of the proposed NO_3_^−^RR mechanism. (a) Online DEMS spectra of NO_3_^−^RR on R‐Co_1_Cu_9_O_X_; Adsorption energy of (b) NO_3_^−^ and (c) NO_2_^−^ at different possible sites on R‐Co_1_Cu_9_O_X_ (Color code: brown for Cu, blue for Co, red for O, light blue for N, light pink for H); (d) Schematic illustration of the proposed self‐adaptive reconstruction and NO_3_^−^RR mechanism; (e) Gibbs free energies of NO_3_^−^RR for different intermediates on Cu, Cu/CoO_X_, and hydroxylated oxidized Cu models, respectively.

Operando electrochemical differential electrochemical mass spectrometry (DEMS) was performed on R‐Co_1_Cu_9_O_X_ to identify the key intermediates and further elucidate the nitrate reduction pathway during the NO_3_^−^RR. As shown in Figure 6a, distinct signals at m/z = 46, 30, 31, 14, 15, 16, and 17 were detected, corresponding to *NO_2_, *NO, *NOH, *N, *NH, *NH_2_, and NH_3_, respectively. These observations provide direct evidence for the formation and sequential transformation of nitrogen‐containing intermediates during nitrate reduction. Combined with the operando Raman and in‐situ ATR‐SEIRAS results, the electrocatalytic NO_3_^−^RR over R‐Co_1_Cu_9_O_X_ is confirmed to proceed through a cooperative catalytic pathway: deoxygenation step (NO_3_^−^→*NO_3_→*NO_2_→*NO_2_H→*NO), hydrogenation step (→*NOH→*N→*NH→*NH_2_ →*NH_3_), and the final desorption of ammonia step [61].

DFT calculations were performed to gain theoretical insight into the reaction mechanism and the reconstructed surface‐localized hydroxyl‐rich Cu species in R‐Co_1_Cu_9_O_X_. Guided by the structural characterization and actual active species analysis, Cu (111), Cu (111)/CoO_X_, and hydroxylated oxidized Cu slabs were adopted as the models (Figures S39–S41). To further clarify the respective roles of Cu related sites and CoO_X_, adsorption‐energy calculations were performed for key NO_3_^−^RR intermediates (Figure 6b,c). Adsorption‐energy calculations reveal that NO_3_^−^ and NO_2_^−^ preferentially adsorb on Cu sites rather than CoO_X_ sites in Cu/CoO_X_. In particular, the self‐adaptively reconstructed surface‐localized Cu–OH‐rich oxidized Cu exhibits the strongest adsorption toward NO_3_^−^ and NO_2_^−^, highlighting their enhanced capability for nitrate capture and activation. In contrast, the comparatively weaker adsorption of NO_2_^−^ on CoO_X_ suggests a catalytic role distinct from that of Cu‐related sites and may facilitate the interfacial conversion of nitrogen‐containing intermediates [50]. NO_3_^−^RR pathways and the corresponding free energy profiles involving adsorption of NO_3_^−^, deoxygenation of *NO_3_, hydrogenation of *N, and desorption of NH_3_ steps, were also investigated to gain theoretical insight into the NO_3_^−^RR mechanism over the actual active species identified in the reconstructed catalytic system, as displayed in Figure 6e. The results were in agreement with previous studies [5, 61]. On pristine metallic Cu, the hydrogenation of *NO_2_ to *NO_2_H is identified as the rate‐determining step, with a free‐energy change of 0.538 eV, indicating the limited ability of Cu to promote the hydrogenation of *NO_2_ intermediates. After introducing CoO_X_, its enhanced water dissociation capability increases the local availability of active hydrogen species (*H), thereby facilitating *NO_2_ hydrogenation and decreasing the corresponding free‐energy barrier to 0.435 eV. Upon self‐adaptive reconstruction, the dynamically generated hydroxylated oxidized Cu surface significantly stabilizes NO_3_^−^ adsorption, rendering the adsorption process more thermodynamically favorable and spontaneous. More importantly, the *NO_2_ → *NO_2_H step becomes thermodynamically downhill on the reconstructed surface, indicating that the hydroxylated oxidized Cu sites effectively regulate the energetics of key intermediates and facilitate the subsequent hydrogenation process, while NH_3_ desorption becomes the dominant energetic limitation (0.910 eV) [28, 61]. These findings collectively suggest that the self‐adaptive reconstruction involving a main reduced metallic Cu domain coupled with surface‐localized Cu–OH‐rich oxidized Cu species is mainly responsible for the initial NO_3_^−^ adsorption and activation while stabilizing key nitrogen‐containing intermediates throughout the hydrogenation pathway. Meanwhile, CoO_X_ primarily facilitates H_2_O dissociation and interfacial *H supply, thereby accelerating hydrogen‐coupled reduction kinetics toward NH_3_ formation. These theoretical results are in good agreement with the experimental observations discussed above, including the strengthened hydrogenation‐related spectroscopic signals and enhanced *H responses revealed by operando characterizations.

As a whole, these findings demonstrated that the superior NO_3_^−^RR performance of Co_1_Cu_9_O_X_ originated from a CoO_X_‐assisted self‐adaptive surface reconstruction process (Figure 6d). Under operating conditions in NO_3_^−^‐containing electrolytes, Cu species dynamically evolve into a Cu–OH‐rich oxidized Cu species surface coupled with reduced metallic Cu domains over extended reaction periods in response to the catalytic environment rather than following a simple unidirectional reduction pathway. Such adaptive surface‐localized hydroxylated oxidized Cu and intrinsic metallic Cu reconstruction from Co_1_Cu_9_O_X_ heterostructure optimized nitrate adsorption and facilitated the stabilization of reaction intermediates through a dynamically regulated interfacial environment. Importantly, Co incorporation as CoO_X_ promoted reversible Cu redox evolution and accelerated surface hydroxylation through strong electronic coupling and more intense interfacial microenvironment change during NO_3_^−^ electroreduction, thereby enabling the balanced generation of catalytically favorable Cu–OH‐rich sites. Moreover, the dual‐phase Cu/CoO_X_ heterogeneous interface modulates the binding affinity toward intermediates and optimizes interfacial *H supply, ensuring precise regulation of the adsorption and hydrogenation of nitrogen‐containing intermediates.

## 3 Conclusion

In summary, Fe, Co, Ni, Ru, and In were selected as the secondary heterogeneous metal candidates in this study to synergistically promote the NO_3_^−^RR performance of CuO. Under electrochemical conditions, the Co_1_Cu_9_O_X_ (CuO/CoO_X_) precatalyst undergoes adaptive reconstruction to form R‐Co_1_Cu_9_O_X_. This adaptively reconstructed heterostructure, mainly composed of Cu/CoO_X_ heterostructure and surface‐localized hydroxylated oxidized Cu species, delivers superior nitrate reduction activity (ammonia yield of 54.68 mg·h^−1^·mg_cat_^−1^, Faradaic efficiency of 95.40% at −0.2 V vs. RHE) compared with other candidates. Such adaptive surface Cu–OH‐rich oxidized Cu and intrinsic metallic Cu reconstruction from Co_1_Cu_9_O_X_ heterostructure enhanced nitrate adsorption and intermediate stabilization, while the Cu/CoO_X_ dual‐phase interface optimized interfacial *H supply for the deep hydrogenation of N‐containing intermediates, thereby achieving high ammonia yield and Faradaic efficiency. These findings highlight the critical role of self‐adapted structural evolution in regulating catalytic performance and offer a generalizable design principle for engineering heterostructures capable of enhanced performance.

## 4 Experimental Section

### 4.1 Materials

Cobalt(II) chloride hexahydrate (CoCl_2_·6H_2_O), nickel(II) chloride hexahydrate (NiCl_2_·6H_2_O), copper(II) chloride dihydrate (CuCl_2_·2H_2_O), indium(III) chloride tetrahydrate (InCl_3_·4H_2_O), iron(II) chloride tetrahydrate (FeCl_2_·4H_2_O), ruthenium(III) chloride n‐hydrate (RuCl_3_·nH_2_O), potassium hydroxide (KOH), potassium nitrate (KNO_3_), sodium nitrite (NaNO_2_), salicylic acid (C_7_H_6_O_3_), trisodium citrate dihydrate (Na_3_C_6_H_5_O_7_·2H_2_O), ammonium chloride (NH_4_Cl, AR), sodium hypochlorite solution (NaClO), dimethyl sulfoxide‐d6 (DMSO‐d6, 99.9%), amidosulfuric acid (H_3_NO_3_S), sodium nitroferricyanide(III) dihydrate (C_5_FeN_6_Na_2_O∙2H_2_O), Nafion perfluorinated resin solution (Nafion, 5 wt.%), hydrochloric acid (HCl, 36.0∼38.0%), sulphuric acid (H_2_SO_4_, 95.0∼98.0%), hydrogen peroxide (H_2_O_2_, 30%), isopropanol (C_3_H_8_O), absolute ethyl alcohol (C_2_H_6_OH), t‐Butyl alcohol ((CH_3_)_3_COH, t‐BuOH, 98.0+%), and deuterium oxide (D_2_O, 99.8%) were purchased from FUJIFILM Wako Pure Chemical Corporation (Japan). N‐1‐Naphthylethylenediamine dihydrochloride (C_12_H_14_N_2_·2HCl), phosphoric acid (H_3_PO_4_), sulfanilamide (C_6_H_8_O_2_N_2_S, 99.0%), and tetramethylammonium hydroxide (TMAOH, (CH_3_)_4_N(OH)) were procured from Sigma‐Aldrich. Ammonium chloride‐^15^N and potassium nitrate (^15^N, 99%) were ordered from Cambridge Isotope Laboratories, Inc. Sodium 1,1,2,2,3,3‐hexadeuterated‐3‐(trimethylsilyl)propane‐1‐sulfonate (DSS) [^1^H NMR standard for D_2_O solvent] was acquired from TCI Chemicals. Nafion 117 proton exchange membrane was purchased from DuPont Co., Ltd. All the chemicals were commercially available and used without purification.

### 4.2 The Preparation of Co_δ_Cu_100‐δ_O_X_ (δ = 0, 5, 10, 15, 20, 50)

A mixed‐metal precursor solution (10 mL, 0.2 M) containing CoCl_2_·6H_2_O and CuCl_2_·2H_2_O was prepared by stirring at room temperature (≈298 K) under ambient conditions. Immediately thereafter, 20 mL of an alkaline oxidizing solution composed of TMAOH (1.0 M, 12 mL), deionized water (6 mL), and 30 wt.% H_2_O_2_ (2 mL) was rapidly introduced. Under vigorous agitation, H_2_O_2_ underwent decomposition with concomitant O_2_ evolution, resulting in the formation of a dark‐brown suspension. The suspension was maintained under continuous stirring at room temperature for 3 h, then isolated by centrifugation, thoroughly rinsed with distilled water, and air‐dried at room temperature for subsequent use. The Co_δ_Cu_100‐δ_O_X_ catalysts with different molar ratios of Co: Cu (5:95, 10:90, 15:85, 20:80, and 50:50) content were prepared with the same method. and corresponding obtained catalyst were noted as Co_1_Cu_19_O_X_, Co_1_Cu_9_O_X_, Co_3_Cu_17_O_X_, Co_1_Cu_4_O_X_, Co_1_Cu_1_O_X_, respectively. The CuO catalyst was prepared by the same method except for the addition of the Co metal precursor (CoCl_2_·6H_2_O).

### 4.3 The Preparation of M_1_Cu_9_O_X_ (M = Fe, Co, Ni, Ru, In)

The Fe_1_Cu_9_O_X_, Ni_1_Cu_9_O_X_, Ru_1_Cu_9_O_X_, and In_1_Cu_9_O_X_ catalysts were synthesized by the same method as Co_1_Cu_9_O_X_. An aqueous solution (10 mL) containing 0.02 M of a metal salt precursor (FeCl_2_·4H_2_O, CoCl_2_·6H_2_O, NiCl_2_·6H_2_O, InCl_3_·4H_2_O, or RuCl_3_·nH_2_O) and 0.18 M CuCl_2_·2H_2_O was stirred and prepared at room temperature (RT, ∼298 K) in air. Subsequently, the same aqueous solution (20 mL) mentioned in the preparation of Co_δ_Cu_100‐δ_O_X_ was added to the metal salt precursor solution rapidly under vigorous stirring. The remaining steps were the same as mentioned above. For other details of the experimental section, please refer to the Supporting Information.

## Author Contributions

**Takeshi Fujita**: conceptualization, supervision, Writing – review and editing, investigation, project administration. **Saikat Bolar**: investigation, validation, formal analysis. **Yongzheng Zhang**: investigation, methodology, formal analysis. **Chunyu Yuan**: investigation, conceptualization, validation, visualization, formal analysis, writing – original draft. **Akitaka Ito**: methodology, investigation, validation, formal analysis. **Tatsuhiko Ohto**: investigation, visualization, formal analysis.

## Conflicts of Interest

The authors declare no conflicts of interest.

## Supporting information




**Supporting File**: advs76573‐sup‐0001‐SuppMat.pdf.

## Data Availability

The data that support the findings of this study are available from the corresponding author upon reasonable request.
