# Supplementary material for: Adaptive Cu Reconstruction in Heterostructure Drives High‐Rate Nitrate‐to‐Ammonia Conversion
Source: Adv Sci (Weinh). 2026 Jul 13:e76573. Online ahead of print. doi: 10.1002/advs.76573 (PMC13360127; doi:10.1002/advs.76573)
Supplement: Supplementary file 1 — Supporting File: advs76573‐sup‐0001‐SuppMat.pdf. [file ADVS-9999-e76573-s001.pdf]

Supporting Information for

Adaptive Cu Reconstruction in Heterostructure Drives High-Rate Nitrate-to-Ammonia Conversion

Chunyu Yuan [a], *Saikat Bolar* [a,b], *Yongzheng Zhang* [c,d], *Akitaka Ito* [a]*, *Tatsuhiko Ohto* [e]* *and Takeshi Fujita* [a,b]*

[a] School of Engineering Science, Kochi University of Technology, 185 Miyanokuchi, Tosayamada, Kami City, Kochi 782-8502, Japan.
[b] Carbon Neutral Materials Center, Kochi University of Technology, 185 Miyanokuchi, Tosayamada, Kami City, Kochi, 782-8502, Japan
[c] Quantum Science Center of Guangdong-Hong Kong-Macao Greater Bay Area, Shenzhen 518102, China
[d] Department of Physics, Southern University of Science and Technology, Shenzhen 518055, China
[e] Graduate School of Engineering, Nagoya University, Furo-cho, Chikusa-ku, Nagoya, Aichi 464-8603, Japan

E-mail:
ito.akitaka@kochi-tech.ac.jp
ohto@nagoya-u.jp
fujita.takeshi@kochi-tech.ac.jp

1. Experimental Section

Catalyst Characterization

Powder X-ray diffraction (XRD) measurements were performed using a Rigaku RINT 2000 diffractometer equipped with a monochromatic Cu Kα radiation source operating at 40 kV and 40 mA. Transmission electron microscopy (TEM) imaging, elemental mapping, and scanning transmission electron microscopy (STEM) were performed using a field-emission transmission electron microscope (JEOL, JEM-2100F, 200 kV). X-ray photoelectron spectroscopy (XPS) measurements were performed using an ESCALAB 250 Xi system or an AXIS Supra$^+$ spectrometer with monochromatized Al Kα excitation and He I radiation, and all binding energies were referenced to the C 1*s* peak at 284.8 eV. Ultraviolet-visible (UV-Vis) absorbance spectra were recorded using a JASCO-V650

spectrophotometer. Raman and in situ Raman spectra were measured using a Horiba LabRAM HR Evolution system equipped with a 532 nm laser. The in situ attenuated total reflection surface-enhanced infrared absorption spectroscopy (ATR-FTIR) technique was conducted using a Bruker VERTEX 80 V spectrometer to investigate the behavior of intermediates. ^{1}H-NMR spectra in H_2O were recorded using a JEOL JMN-ECZ400S spectrometer (399.78 MHz) under the no-deuterium (No-D) conditions.

Electrochemical Measurements

All electrochemical measurements were conducted using an Ivium-Stat electrochemical analyzer (Ivium Technologies, Netherlands). NO_3^-RR measurements were performed in a commercial H-type electrolytic cell separated by a Nafion 117 membrane. A three-electrode system was used for electrochemical measurements. The catalyst ink was prepared by mixing 5.0 mg of catalyst with 720 μL of isopropanol, 240 μL of deionized water (DI), and 40 μL of a 5 wt% Nafion solution, followed by sonication for 60 min in a low-temperature ice-water bath to form a homogeneous ink. The working electrode was prepared by dropping 20 μL of catalyst ink onto a 1 × 1 cm^2 carbon paper. Before NO_3^- RR measurements, the catalyst was pre-activated by cyclic voltammetry (CV) cycling within a potential range from 0.3 V to − 0.7 V in a 1.0 M KOH solution for 50 cycles, followed by 1 h of electrolysis in 1.0 M KOH containing 100 mM KNO_3. Linear sweep voltammetry (LSV) curves were recorded at a scan rate of 10 mV s^{-1}. Electrochemical impedance spectroscopy (EIS) tests were performed at an operating potential over a frequency range from 100 kHz to 0.01 Hz with an amplitude of 10 mV. Chronoamperometry tests were performed in a 1.0 M KOH solution containing different concentrations of KNO_3 at various potentials for 1 h. For isotope labeling experiments, $K^{14}NO_3$ and $K^{15}NO_3$ were used as the N-source for electrocatalytic NO_3^- RR. For the electrochemical surface area (ECSA) study, cyclic voltammetry (CV) curves were measured at different scan rates (20~120 mV s^{-1}) within the non-Faradic potential range from 0.92 V to 1.02 V vs. RHE. The capacitive current density ($\Delta j = (j_{anodic} - j_{cathodic})/2$) was plotted against the scan rate (ν), and the slope of the linear fit corresponds to C_{dl},. The ECSA was calculated according to the equations: ECSA = C_{dl}/C_s, while the resulting ECSA is expressed in cm^2, where C_{dl} is the double-layer capacitance and C_s is the specific capacitance, assumed to be 40 μF cm^{-2}. Possible gas products such as H_2 and N_2 were detected using a gas chromatograph (Shimadzu GC-2014) equipped with a thermal conductivity detector. To evaluate the rate constant, chronoamperometry tests were performed at -0.2 V vs RHE in 1 M KOH containing 100 mM NO_3^- or 100 mM NO_2^-. The rate constants K_1 (for NO_3^- reduction) and K_2 (NO_2^- reduction) were determined by monitoring the concentration evolution of NO_3^- or NO_2^- as a function of electrolysis time,

assuming exponential decay according to first-order kinetics [1]. The values of K_1 and K_2 were calculated using: $C_t = C_0 * \exp(-k * t)$ where C_0 is the initial concentration of NO_3^- or NO_2^- and C_t is the concentration of NO_3^- or NO_2^- at the electrocatalysis time t.

Determination of ion concentration

Quantification of NH_4^+

The NH_4^+ generated in the electrolyte was determined using the indophenol blue method and nuclear magnetic resonance (NMR) spectroscopy [2]. For the indophenol blue method, 2 mL of appropriately diluted electrolyte with an NH_4^+ concentration within the detection limit range was pipetted and mixed with 2 mL of 1 M NaOH solution containing sodium citrate (5 wt. %) and salicylic acid (5 wt. %), followed by the addition of 1 mL of 0.05 M sodium hypochlorite solution and 200 μL of sodium nitroferricyanide (1 wt%). After incubation in the dark for 2 h, the absorbance at 655 nm of all chromogenic solutions was measured using the UV-vis spectrometer. The concentration–absorbance and calibration curves were obtained using a series of standard ammonia chloride solutions at different concentrations in 1 M KOH. Further, 1 mL of the electrolyte after electrolysis was acidified with 4.0 M H_2SO_4 to adjust the pH to 1–2. Then, 900 μL of the obtained solution was mixed with 30 μL of D_2O and 70 μL of sodium 1,1,2,2,3,3-hexadeuterated-3-(trimethylsilyl)propane-1-sulfonate (DSS, 20 mM), which was used as an internal standard for chemical shift referencing at the horizontal axis (0.00 ppm) and signal integration. The detection method for standard $^{15}NH_4^+$ and $^{14}NH_4^+$ was identical to that aforementioned.

Quantification of NO_2^-

The NO_2^- byproducts were determined using a modified Griess method [3]. To obtain the chromogenic agent, 0.04 g of $C_{12}H_{14}N_2 \cdot 2HCl$, 0.8 g of $C_6H_8O_2N_2S$, and 2.0 mL of an 85% H_3PO_4 solution were dissolved in 10.0 mL of DI water. Further, 5 mL of diluted post-electrolysis electrolytes were mixed with 0.1 mL of the chromogenic agent. After storage in the dark for 20 min, the absorbance at 540 nm of the solution was measured using the UV-vis spectrometer. The concentration–absorbance and calibration curves were constructed using a series of standard sodium nitrite solutions at different concentrations in 1 M KOH.

Quantification of N_2H_4

The generation and amount of N_2H_4 were determined using the Watt and Chrisp method[4]. Typically, a chromogenic agent consisting of concentrated HCl (10 mL),

ethanol (100 mL), and 4-(dimethylamino) benzaldehyde (2.0 g) was prepared. Subsequently, 2.0 mL of the diluted electrolyte was mixed with 2.0 mL of the prepared chromogenic agent. The mixture solution was incubated in the dark at 25 °C for 15 min prior to UV-vis adsorption measurements, and the absorbances at 460 nm were then recorded. The concentration–absorbance and calibration curves with appropriate $N_2H_4{\cdot}H_2O$ concentrations were obtained using a series of standard ammonia chloride solutions at different concentrations in 1 M KOH.

Quantification of NO_3^-

The concentration of NO_3^- was determined with the following procedure[5]: after electrocatalysis, 5.0 mL of diluted electrolyte at an appropriate concentration was mixed with 0.1 mL of a 1.0 M HCl solution and 0.01 mL of a 0.8 wt% H_3NO_3S solution. After standing at room temperature for 20 min, the absorbance intensities at 220 nm and 275 nm were measured using the UV-vis spectrometer. The NO_3^- concentration was calculated using the absorbance defined as $A = A_{220nm} - 2 \times A_{275nm}$ in conjunction with the calibration curve. The concentration–absorbance and calibration curves were obtained using a series of standard KNO_3 solutions at different concentrations in 1 M KOH.

Calculation Methods

The NH_3 yield rate, NH_3 FE, NO_2^- yield rate, and NO_2^- FE were determined as:

$$NH_3 \text{ yield rate} = \frac{C_{NH_4^+} * V}{S * t}$$

$$NH_3 \text{ FE} = \frac{C_{NH_4^+} * V * 8 * F}{17 * Q}$$

$$NO_2^- \text{ yield rate} = \frac{C_{NO_2^-} * V}{S * t}$$

$$NO_2^- \text{ FE} = \frac{C_{NO_2^-} * V * 2 * F}{46 * Q}$$

where $C_{NH_4^+}$ and $C_{NO_2^-}$ represent the concentrations of NH_4^+ and NO_2^- in the electrolyte (μg mL^{-1}), respectively ; V refers to the volume of electrolyte (60 mL); S is the geometric area of electrode (cm^2); t is the time of the chronoamperometry test (1 h); F is the Faraday constant (96485 C mol^{-1}); 8 represents the number of electrons transfer from NO_3^- to NH_3; 17 is the molecular mass of NH_3; Q stands for the total charge amount during electrocatalysis, which was integrated from the *i*–*t* curve; 2 represents the number of electrons transfer from NO_3^- to NO_2^-; and 46 is the molecular mass of NO_2^-.

Operando EPR experiments

The experiments of *H capture were conducted in electrolytes of 1.0 M KOH with and without NO_3^- (Bruker A300). In order to ensure sufficient *H generation, the cathode area was set to 2 × 2 $cm^{2,}$ and the electrolyte was set to 30 mL. After electrolysis at −0.2 V versus RHE for 10 min, 20 μL of electrolyte was withdrawn, immediately mixed with an equal volume of DMPO solution, and transferred into a capillary tube for EPR analysis.

In situ Raman measurement

In situ Raman spectroscopy was detected using a confocal Raman microscope (Horiba LabRAM HR Evolution) with a 532 nm single longitudinal-mode laser at room temperature. An in situ electrochemical cell (K008) manufactured by Tianjin Aida Co., Ltd. was selected to fit the Raman spectrometer to perform the measurement. An ink was prepared by mixing 5 mg of catalyst, 40 μL of Nafion solution, 720 μL of isopropanol, and 240 μL of deionized water, and was uniformly coated onto carbon paper. The electrocatalytic NO_3^-RR on samples was performed in an in situ Raman electrochemical three-electrode cell filled with Ar-saturated 1.0 M KOH+100 mM KNO_3. The chronoamperometric tests for 10 min from 0.2 to -0.5 V vs. RHE were carried out on a CHI 760E electrochemical workstation. In addition, in situ Raman spectra were collected at −0.2 V vs. RHE, where the catalyst exhibits the highest FE, over different reaction durations to investigate the reconstruction behavior.

In situ ATR-FTIR measurement

In situ ATR-FTIR was conducted on Bruker VERTEX 80v. An ink was prepared by mixing 5 mg of catalyst, 40 μL of Nafion solution, 720 μL of isopropanol, and 240 μL of deionized water, and was uniformly coated onto germanium crystal. The germanium crystal was embedded in an electrochemical three-electrode cell as the working electrode with an Ag/AgCl reference electrode and a platinum-wire counter electrode (EC-ATR-H). The measurements were all obtained by 120 scans at a spectral resolution of 8 cm^{-1}. Ar-saturated 1.0 M KOH+ 100 mM KNO_3 solution was flowed as electrolyte. The chronoamperometric tests from 0.2 to -0.5 V vs. RHE were carried out on a CHI 760E electrochemical workstation, accompanied by the spectrum collection. All spectra were collected after background subtraction. The relevant spectral curves were acquired within 10 min during i–t measurements. All spectra were presented in absorbance units as $-\log(R/R_0)$, where R and R_0 represent the intensities of the reflectance of the sample spectrum and the background spectrum, respectively.

Online DEMS measurements

Online differential electrochemical mass spectrometry (DEMS) measurement was performed with a mass spectrometer (Linglu QMG220) and an electrochemical workstation. The electrochemical cell comprises a catalyst working electrode, Ag/AgCl reference electrode, and Pt wire counter electrode, which was working in an electrolyte containing Ar-saturated 1 M KOH + 100 mM KNO_3 solution. The DEMS measurement was conducted at constant potential -0.2 V vs. RHE with 0.8 mL/min continuous argon purging, and the corresponding mass signals were detected during this period. During the measurement, the mass spectrometer signals were allowed to return to the baseline level. Mass signals were collected in quadruplicate under identical conditions to ensure reproducibility.

Density functional theory calculations

To investigate the free energy diagram of NO_3^- reduction, density functional theory (DFT) calculations were performed using the VASP code with the projected augmented wave method and the Perdew–Burke–Ernzerhof exchange-correlation functional[6–8]. The plane-wave energy cutoff was set to 400 eV. Grimme's D3(BJ) scheme was employed for dispersion correction to accurately estimate the adsorption energies of the intermediates[9,10].

The Cu(111) surface was approximated using a p(4×4) slab model containing four Cu layers and more than a 20 Å vacuum region. The Cu lattice was expanded by 5.8% to make it commensurate with a p(1×1) CoO(111) slab model and thereby adapt to the Cu(111)/CoO(111) interface model. This slight expansion of the Cu(111) lattice did not significantly affect the energy diagram. The CoO cluster consisted of three formula units of Co_3O_4. Hydroxylated oxidized Cu was simulated using a defective $Cu(OH)_2$(021) surface represented by a p(4×1) slab model containing four layers. Partial dehydroxylation of the top layer was introduced to generate exposed Cu sites, mimicking the dynamically reconstructed hydroxylated Cu surface under reaction conditions. The k-points were sampled using a (2×2×1), (2×2×1), and (1×1×1) meshes for Cu(111), Cu(111) with the CoO cluster, and hydroxylated oxidized Cu (021) surfaces, respectively. The free energy of the adsorption of each intermediate was calculated as:

$$\Delta G = \Delta E_{ads} + \Delta E_{ZPE} + \Delta H_{0-T} + T\Delta S$$

where ΔE_{ads} is the total energy difference of reactants and adsorbed system with reference to H_2(g), NO(g), and H_2O(g), ΔE_{ZPE} is the zero-point energy of the vibrations of the adsorbate, $\Delta H_{0-\mathrm{T}}$ is the enthalpy difference between 0 K and T K, and S is the

entropy.

2. Results and Discussion

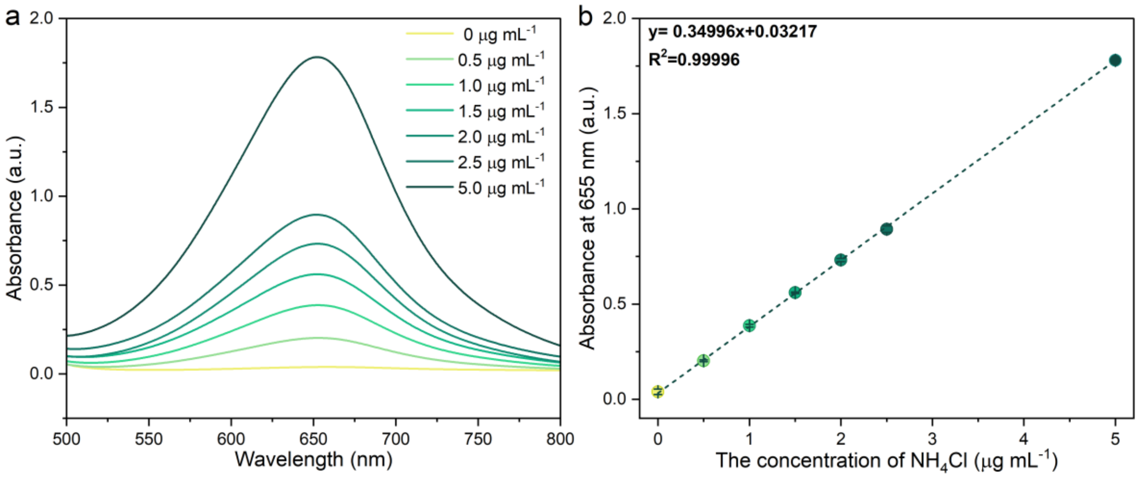

Figure S1. Standard calibration of NH_4^+ quantification. (a) UV-vis absorption spectra for NH_4^+ detection and (b) the corresponding standard calibration curve for NH_4^+ determined at 655 nm in 1 M KOH with the indophenol blue method.

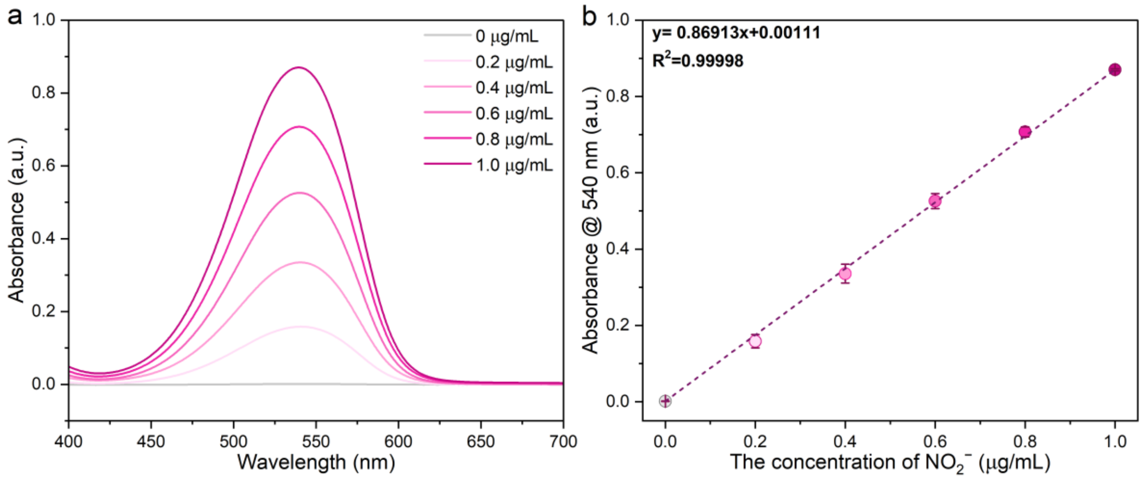

Figure S2. Standard calibration of nitrite quantification. (a) UV-vis absorption spectra of nitrite detection and (b) the corresponding standard calibration curve for nitrite determined at 540 nm in 1 M KOH using the N-(-1-naphthyl)-ethylenediamine dihydrochloride spectrophotometric method.

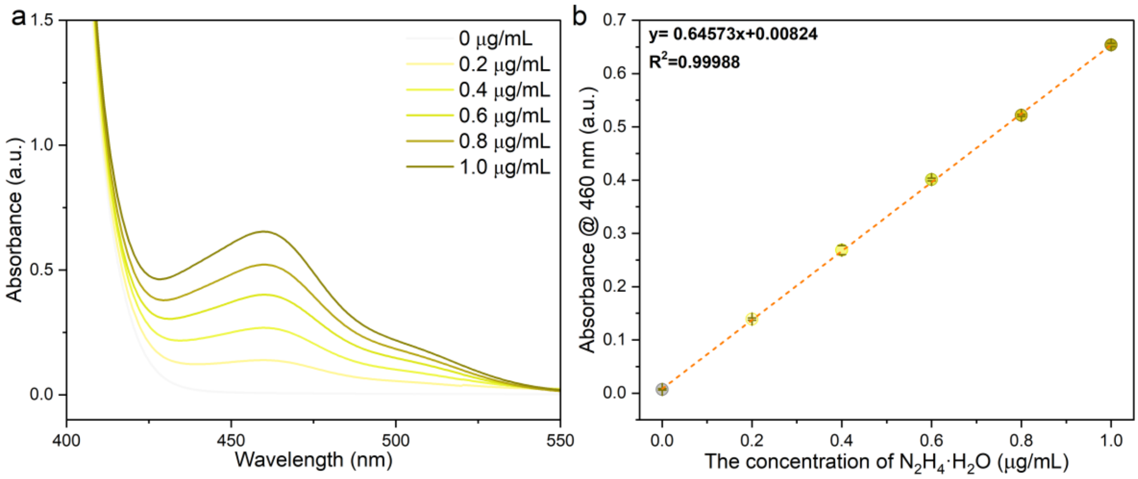

Figure S3. Standard calibration of hydrazine quantification. (a) UV-vis absorption spectra of hydrazine detection and (b) the corresponding standard calibration curve for hydrazine determined at 460 nm in 1 M KOH using the Watt and Chrisp method.

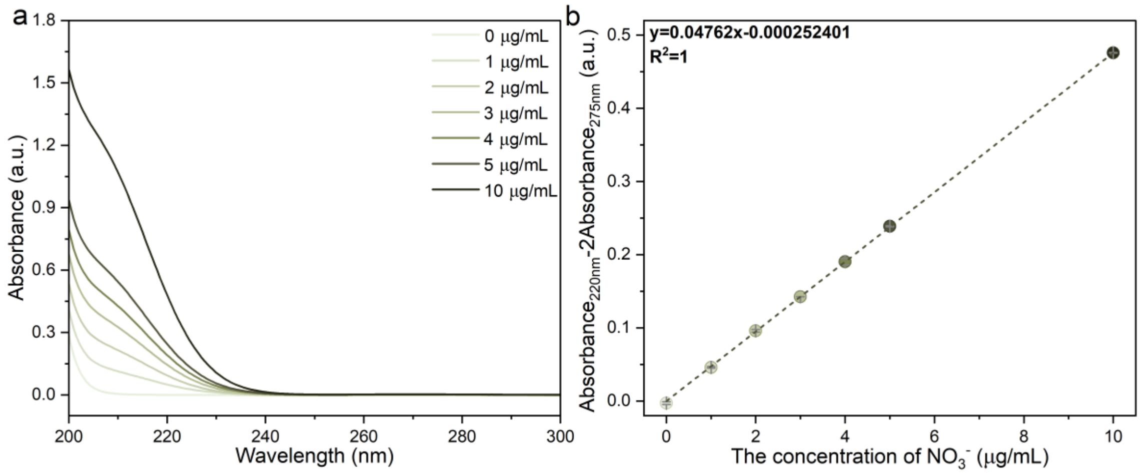

Figure S4. Standard calibration of nitrate quantification. (a) UV-vis absorption spectra of nitrate detection. (b) Standard calibration line for nitrate, determined at $Absorbance_{220nm}{-}2Absorbance_{275nm}$.

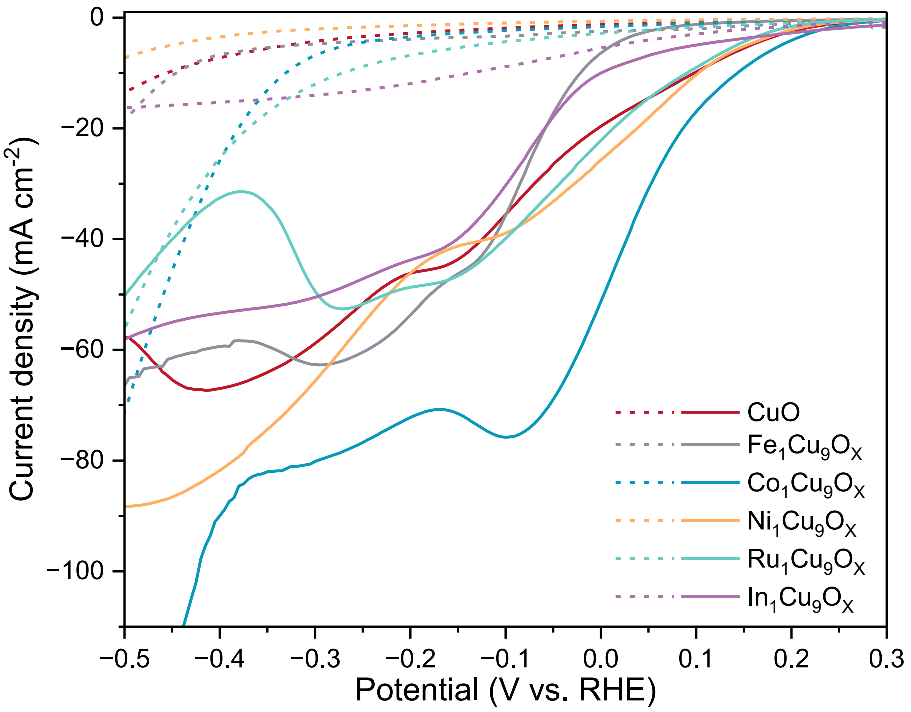

Figure S5. LSV curves of Cu-based $M_1Cu_9O_X$ (M is one of Fe, Co, Ni, Ru, In elements) catalysts after pre-activation, at a scan rate of 10 mV s^{-1} in 1 M KOH solution without (dotted line) and with (solid line) 100 mM NO_3^-.

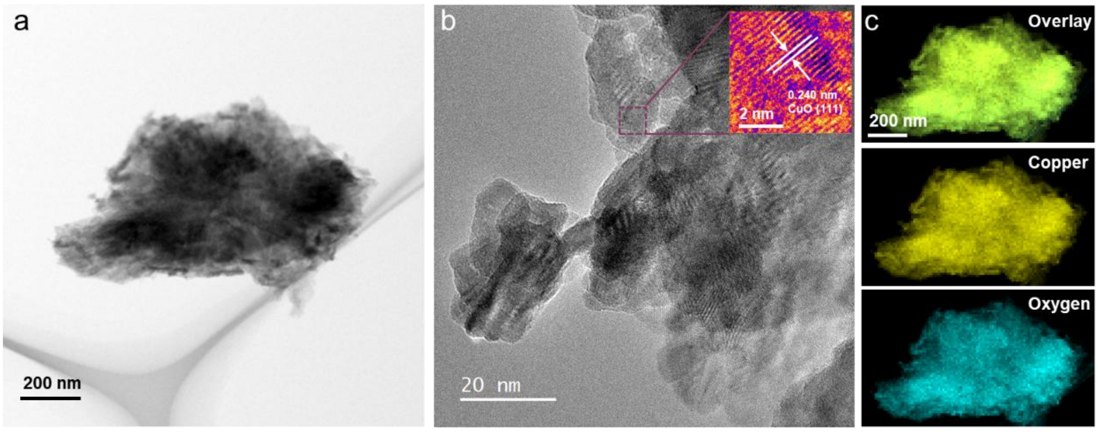

Figure S6. (a) TEM and (b) HR-TEM image, (c–e) Corresponding elemental mapping of CuO.

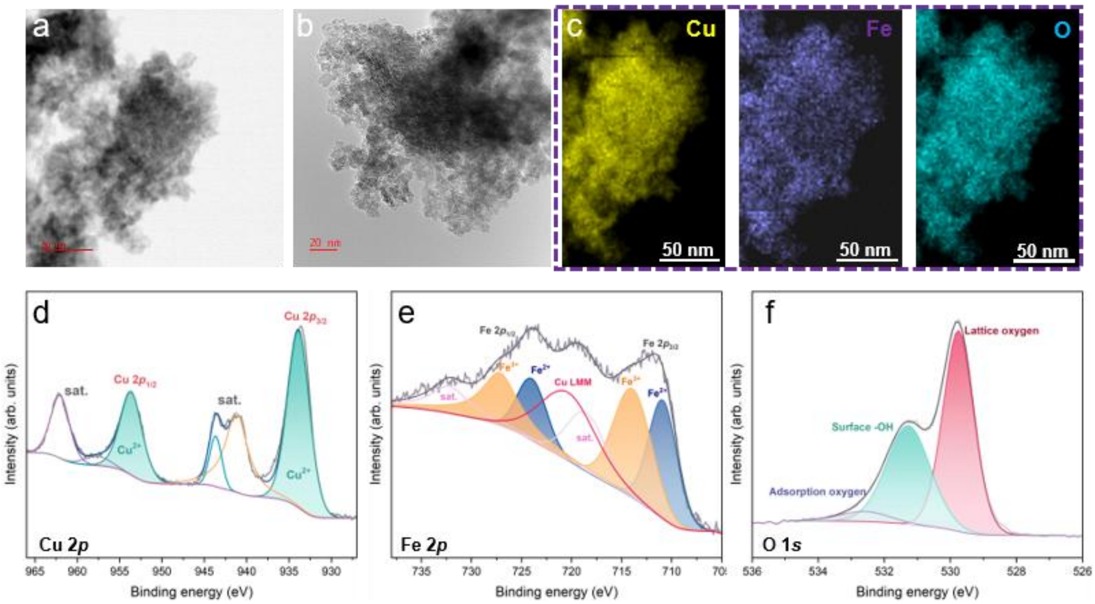

Figure S7. (a) STEM image, (b) HRTEM image, and (c) corresponding STEM elemental maps of $Fe_1Cu_9O_X$; High-resolution XPS spectra of $Fe_1Cu_9O_X$: (d) Cu 2*p*, (e) Fe 2*p*, and (f) O 1*s*.

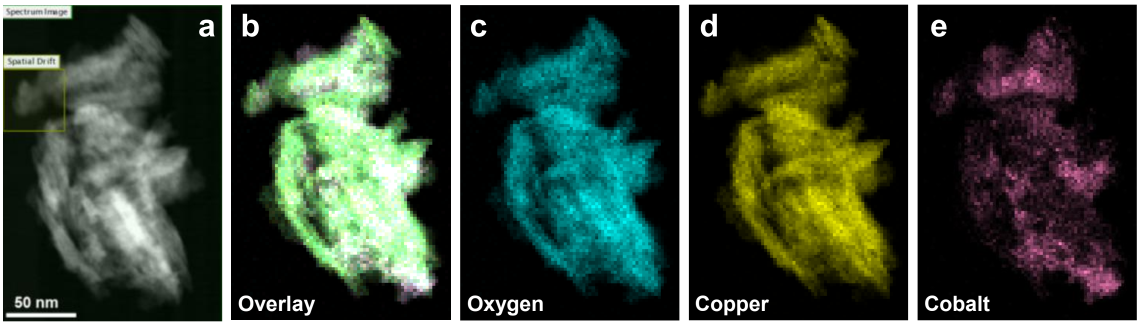

Figure S8. (a) STEM image. (b–e) Corresponding elemental mapping of $Co_1Cu_9O_X$.

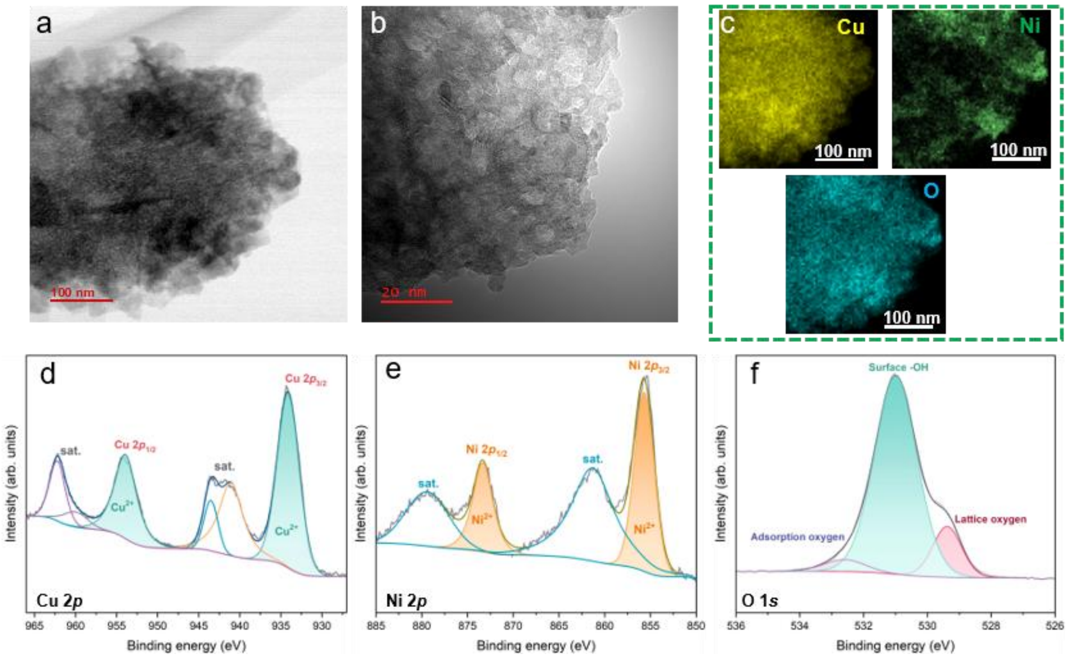

Figure S9. (a) STEM image, (b) HRTEM image, and (c) corresponding STEM elemental maps of $Ni_1Cu_9O_X$; High-resolution XPS spectra of $Ni_1Cu_9O_X$: (d) Cu 2*p*, (e) Ni 2*p*, and (f) O 1*s*.

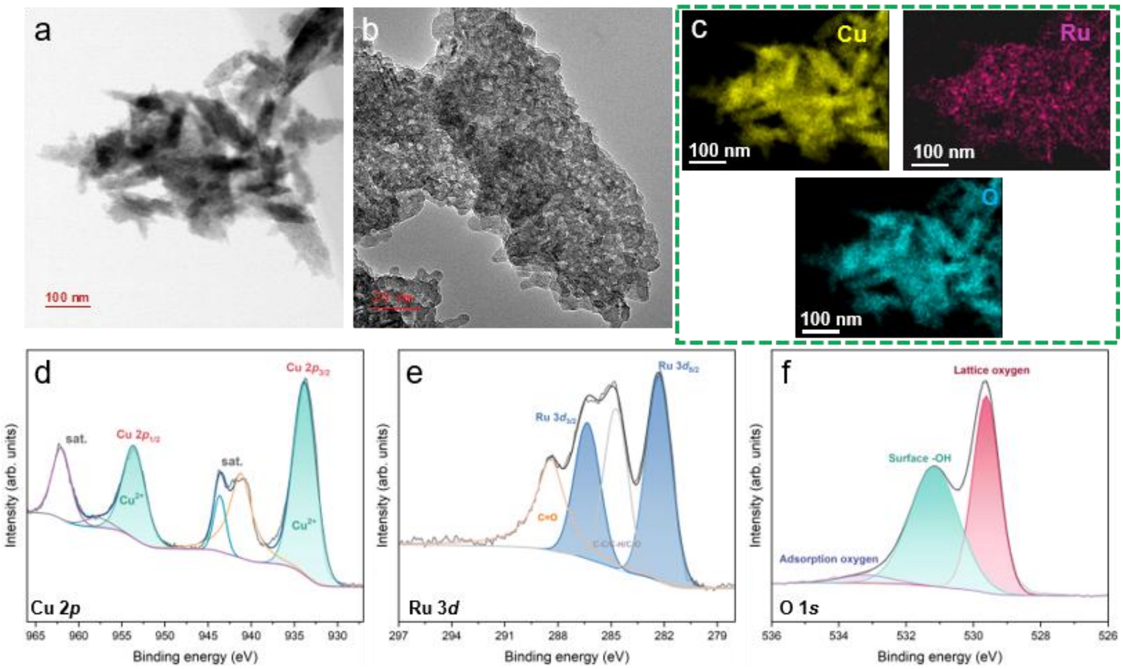

Figure S10. (a) STEM image, (b) HRTEM image, and (c) corresponding STEM elemental maps of $Ru_1Cu_9O_X$; High-resolution XPS spectra of $Ru_1Cu_9O_X$: (d) Cu 2*p*, (e) Ru 3*d*, and (f) O 1*s*.

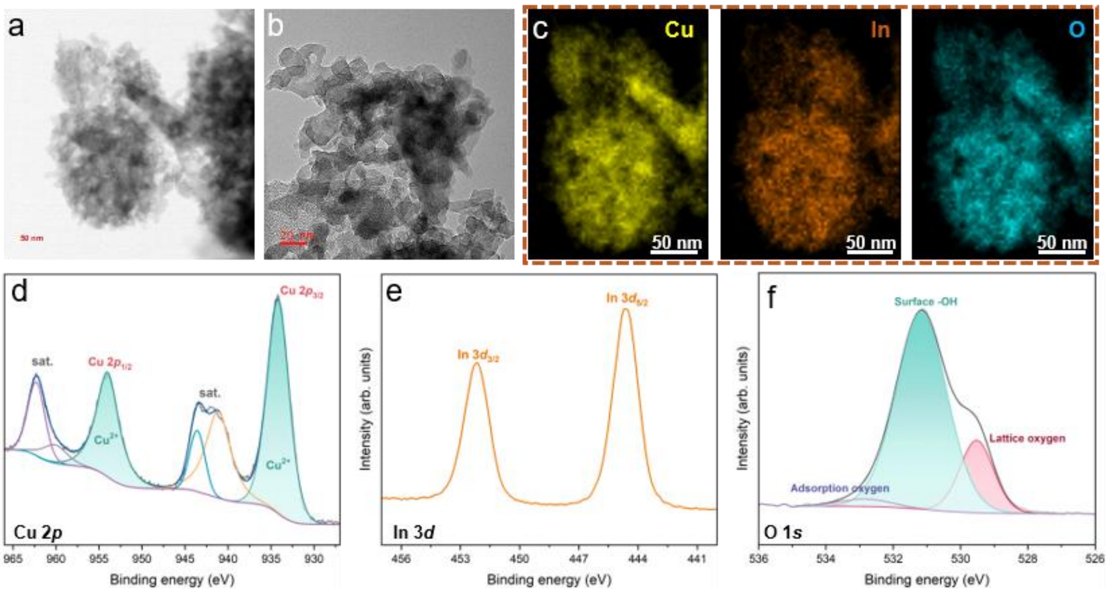

Figure S11. (a) STEM image, (b) HRTEM image, and (c) corresponding STEM elemental maps of $In_1Cu_9O_X$; High-resolution XPS spectra of $In_1Cu_9O_X$: (d) Cu 2*p*, (e) In 3*d*, and (f) O 1*s*.

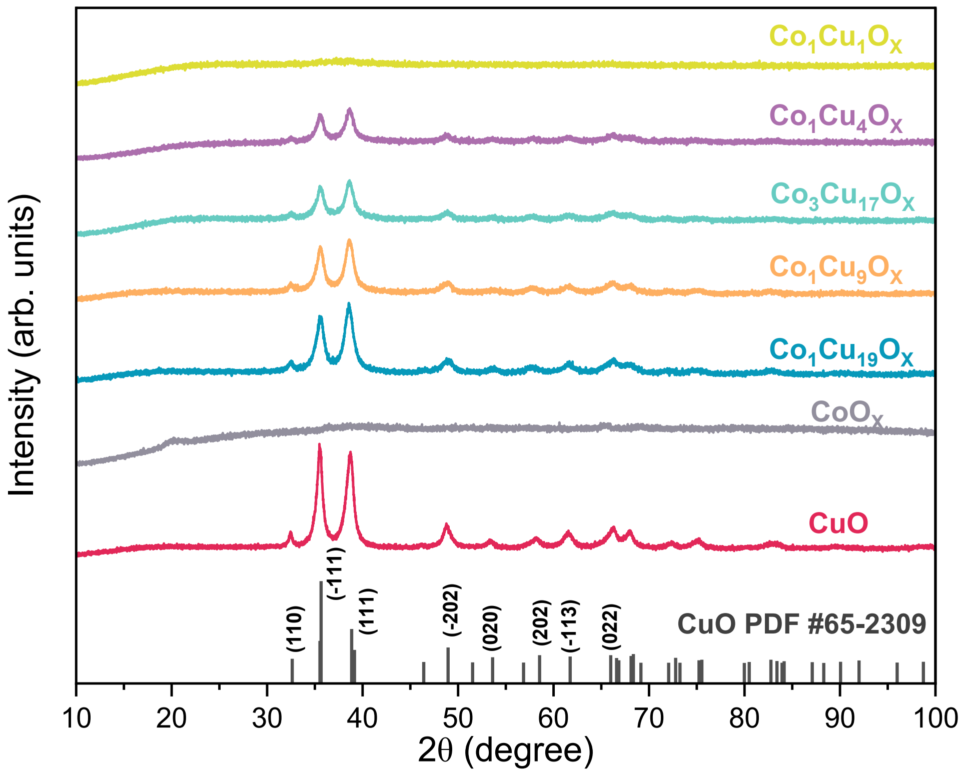

Figure S12. XRD patterns of CoO_X and $Co_{\delta}Cu_{100-\delta}O_X$ (δ = 0, 5, 10, 15, 20, 50, simplified stoichiometric ratio representation) composites with different feeding molar ratios.

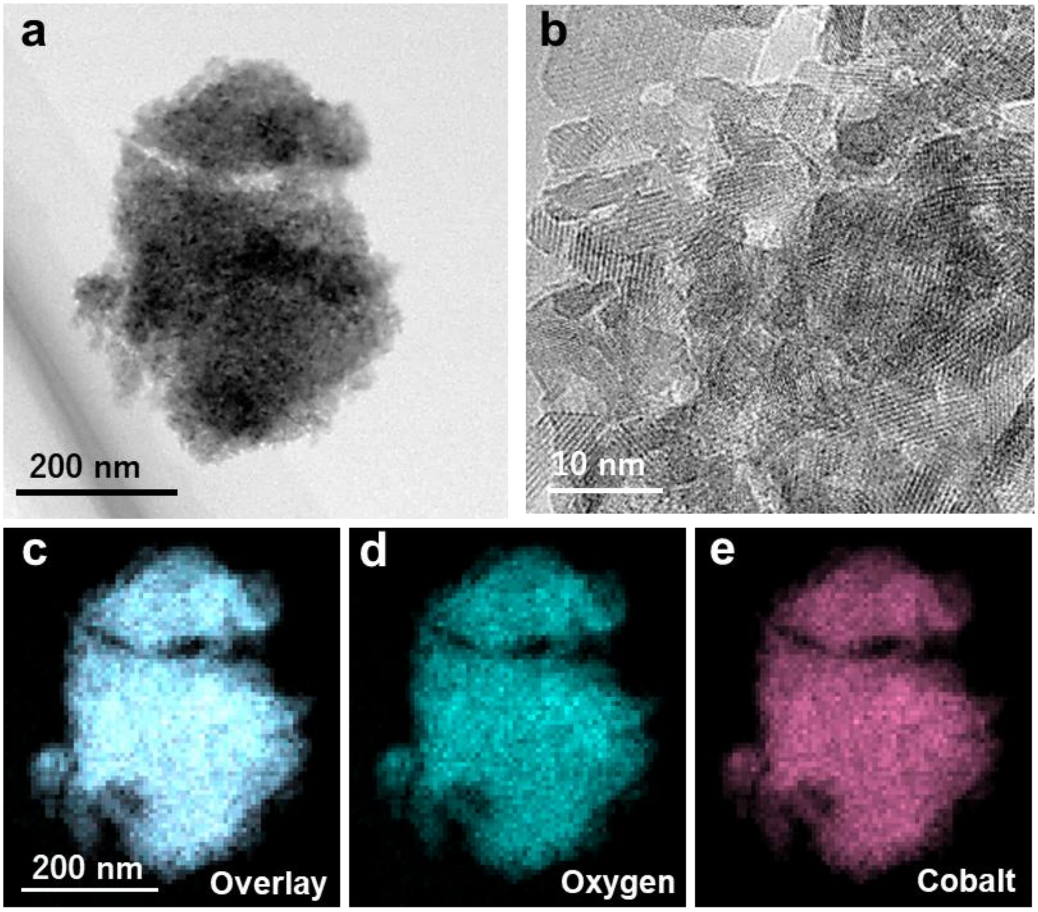

Figure S13. (a) TEM and (b) HR-TEM image, (c–e) Corresponding elemental mapping of CoO_X.

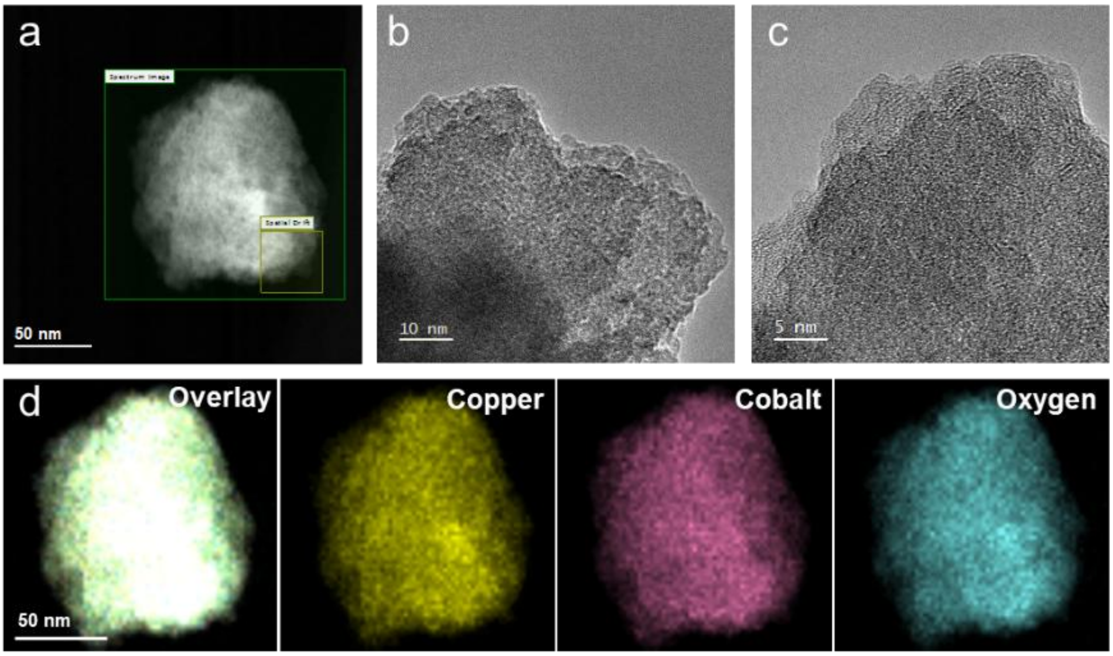

Figure S14. (a) STEM image, (b–c) HR-TEM images, and (d) corresponding elemental mapping in the selected region in (a) of $Co_1Cu_1O_X$.

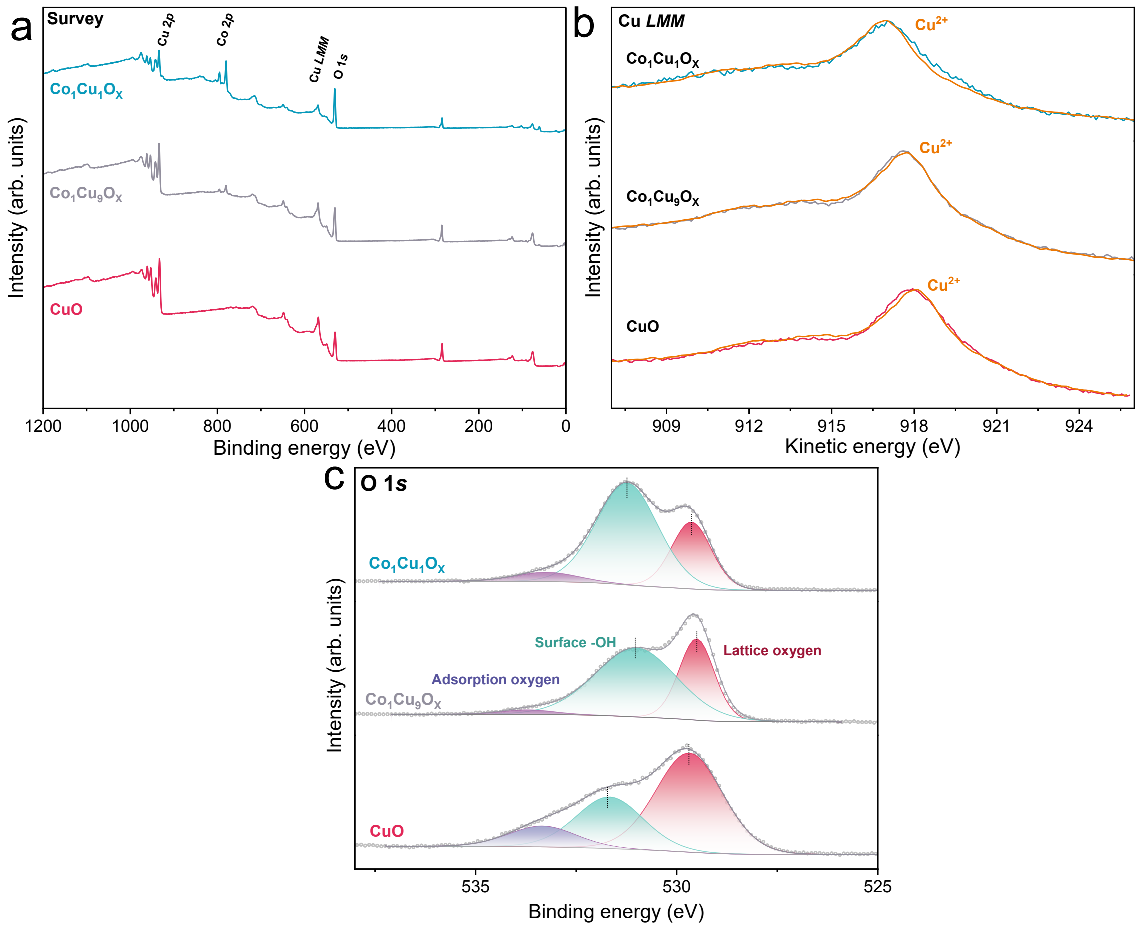

Figure S15. (a) XPS wide survey spectra. (b) Cu LMM spectra with NLS-based curve fitting. (c) High-resolution XPS spectra of O 1*s* in CuO_X, $Co_1Cu_9O_X$, and $Co_1Cu_1O_X$.

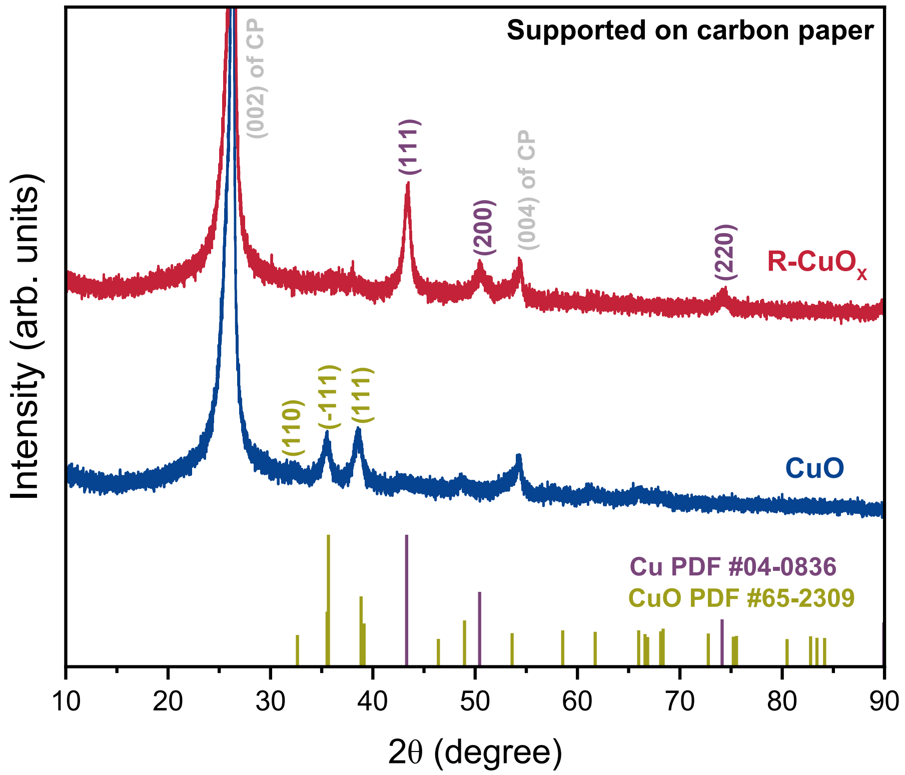

Figure S16. XRD pattern of CuO and R-CuO$_X$ (CuO after pre-activation) supported on carbon paper (pre-activation: 50 cycles of CV activation from 0.3 V to −0.7 V vs. RHE in 1 M KOH and 1 h of electrolysis in 1 M KOH+100mM KNO_3.)

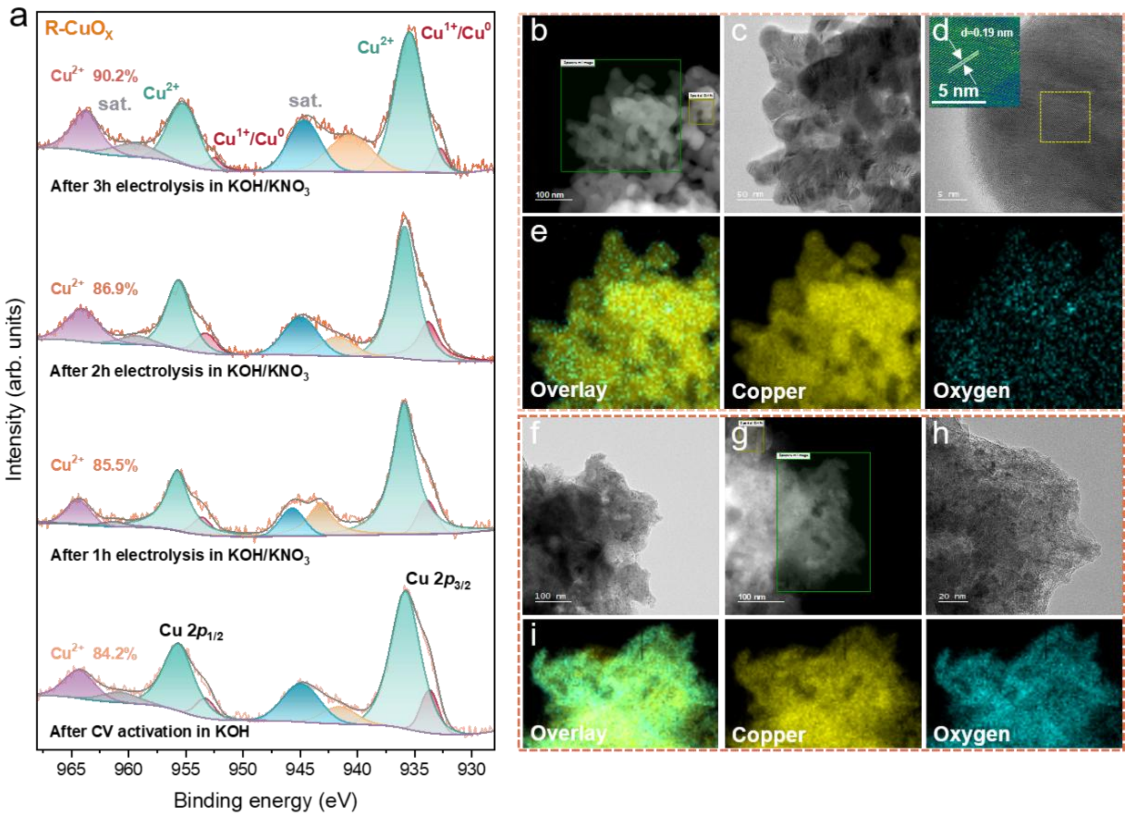

Figure S17. **Morphological and structural characterization of reconstructed CuO.** (a) Quasi operando XPS analyses of Cu 2*p* in CuO after CV activation in KOH and after both CV activation in KOH and chronoamperometric electrolysis in KOH+KNO_3 electrolyte with different durations for 1, 2, and 3 h. (b–e) HR-TEM, HAADF-STEM, and corresponding EDS element map of the marked region of the reconstructed CuO catalyst after CV activation in KOH. (f–i) HR-TEM, HAADF-STEM, and corresponding EDS element map of the marked region of the reconstructed CuO catalyst after both CV activation in KOH and 2 h electrolysis in KOH/KNO_3 electrolyte.

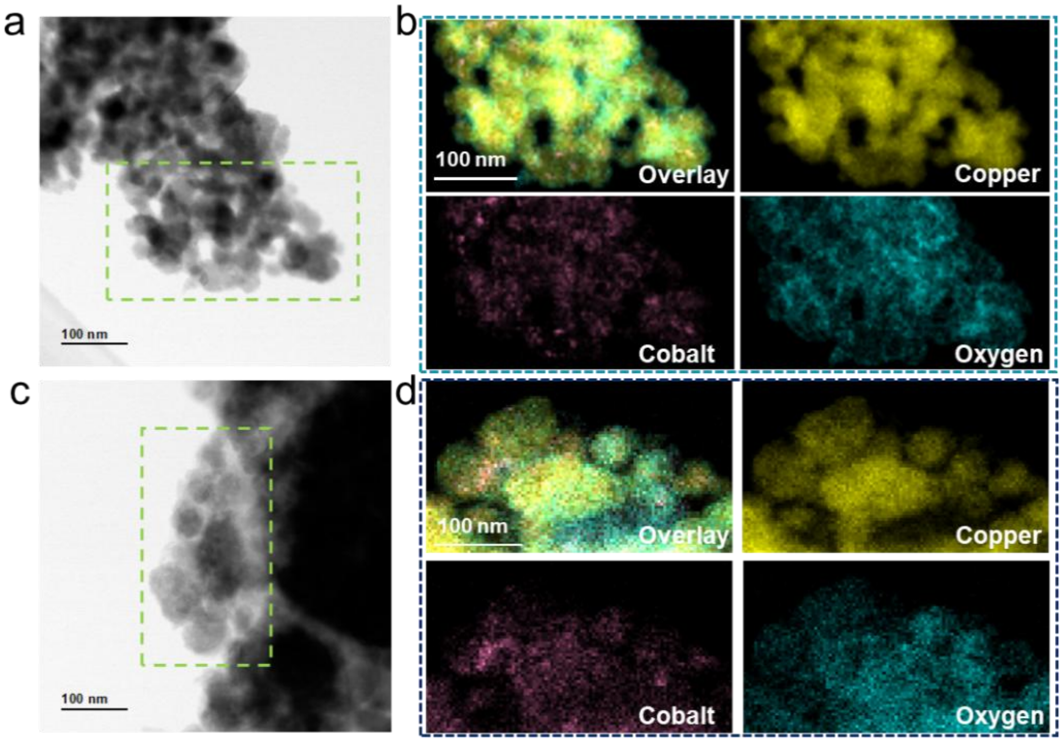

Figure. S18. Morphological and structural characterization of reconstructed $Co_1Cu_9O_X$. (a, b) HAADF-STEM image and corresponding EDS element map of the marked region of $Co_1Cu_9O_X$ catalyst after CV activation in KOH. (c, d) HAADF-STEM image and corresponding EDS element mappings of the marked region of $Co_1Cu_9O_X$ catalyst after both CV activation in KOH and 2 h of electrolysis in KOH+KNO_3 electrolyte.

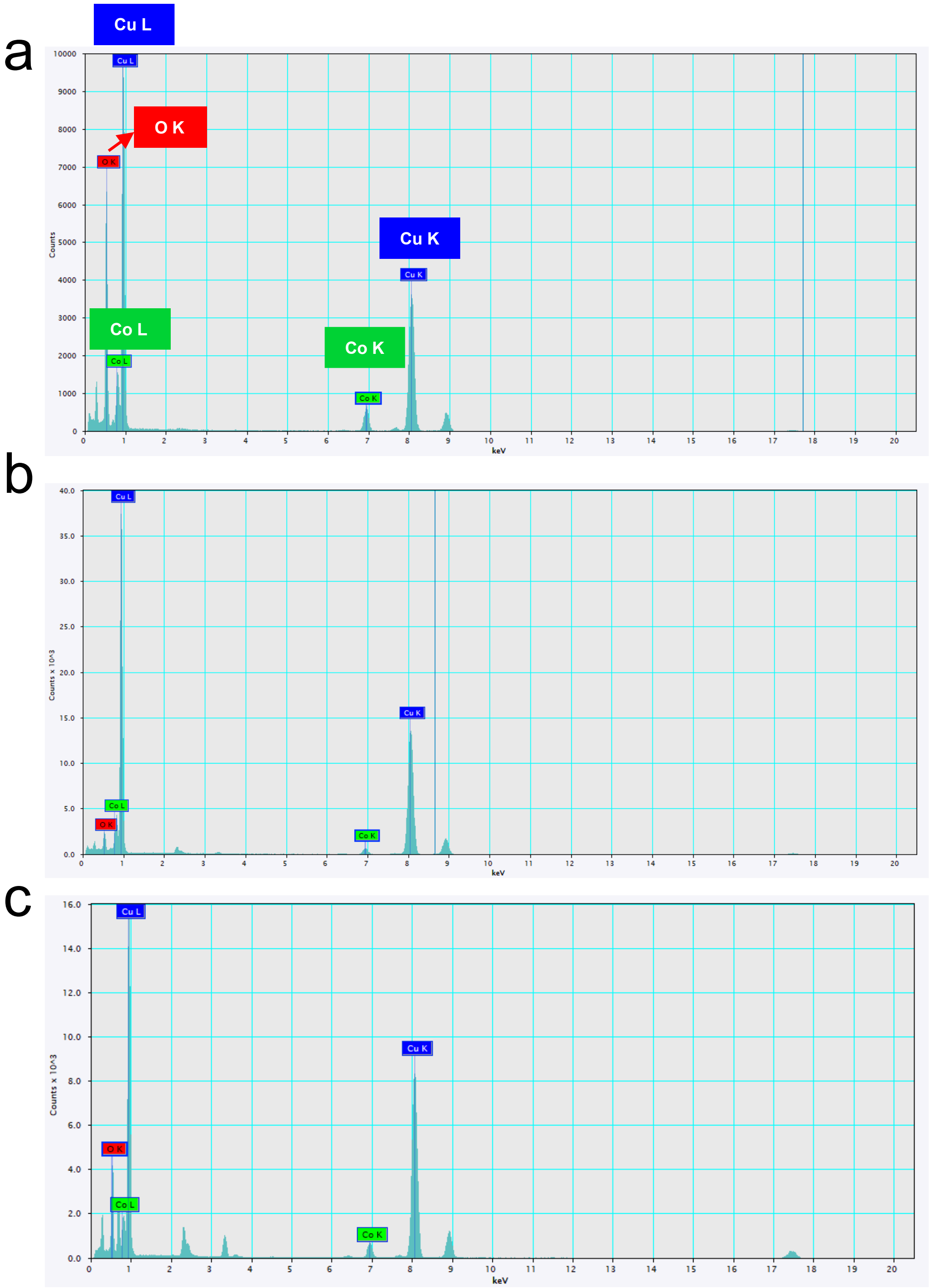

Figure S19. EDS spectra of (a) original $Co_1Cu_9O_X$, (b) $Co_1Cu_9O_X$ after CV activation in KOH electrolyte, and (c) $Co_1Cu_9O_X$ after both CV activation in KOH and chronoamperometric electrolysis for 2 h in $KOH+KNO_3$ electrolyte.

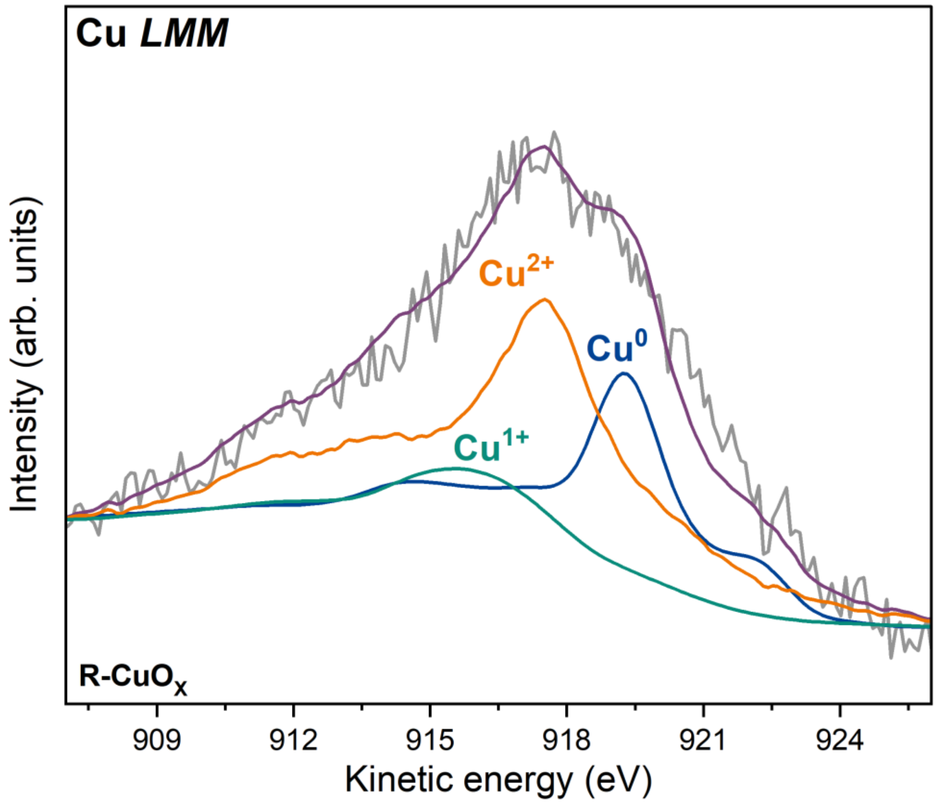

Figure S20. Cu LMM XPS spectra with NLS-based curve fitting of reconstructed CuO (R-CuO_X), pre-activated by cyclic voltammetry (CV) cycling within a potential range from 0.3 V to − 0.7 V in a 1.0 M KOH solution for 50 cycles, followed by 1 h of chronoamperometric electrolysis in 1.0 M KOH containing 100 mM KNO_3.

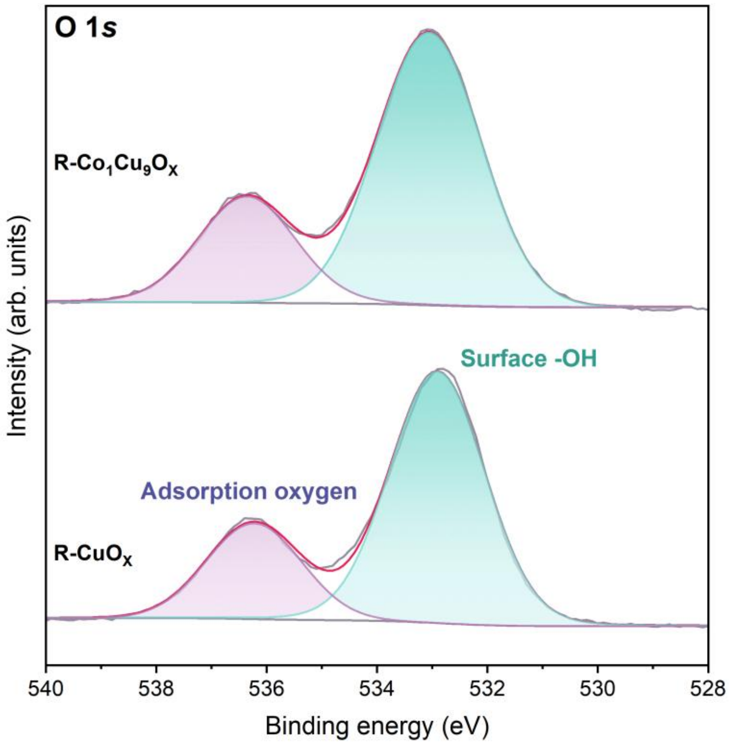

Figure S21. High-resolution XPS spectra of O 1*s* in reconstructed CuO (R-CuO_X) and $Co_1Cu_9O_X$ (R-$Co_1Cu_9O_X$), pre-activated by cyclic voltammetry (CV) cycling within a potential range from 0.3 V to − 0.7 V in a 1.0 M KOH solution for 50 cycles, followed by 1 h of chronoamperometric electrolysis in 1.0 M KOH containing 100 mM KNO_3.

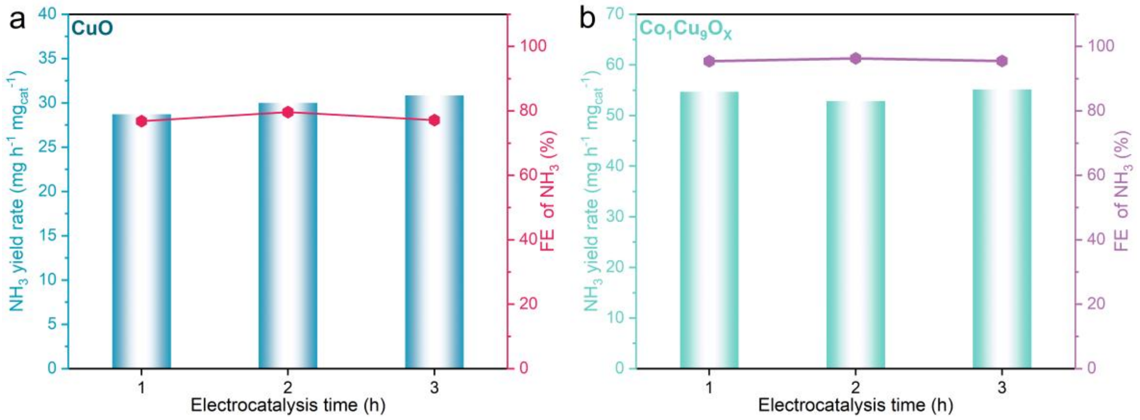

Figure S22. The comparison of NH_3 yield rate and FEs of NH_3 on (a) CuO and (b)$Co_1Cu_9O_X$ after cyclic voltammetry (CV) in 1.0 M KOH and subsequently subjected to 1h, 2h, 3h of chronoamperometric electrolysis for NO_3^-RR at −0.2 V vs. RHE.

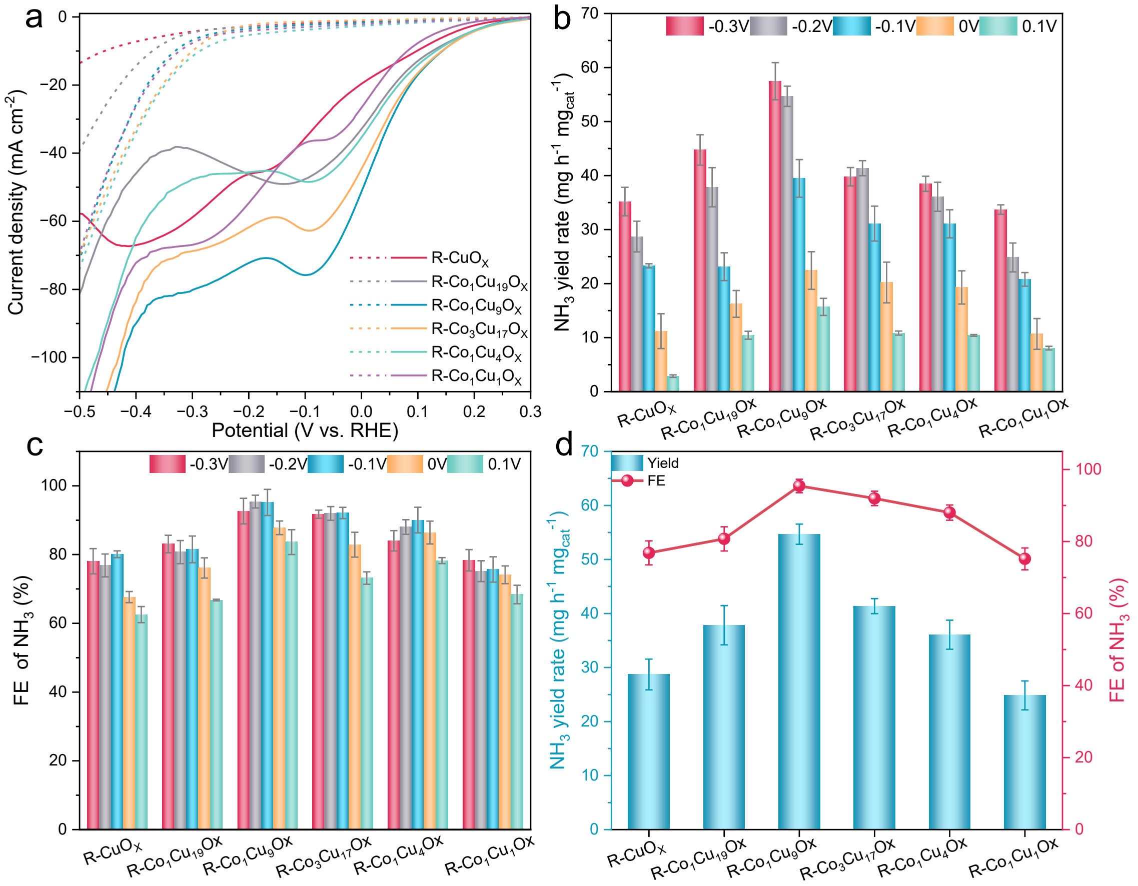

Figure S23. **Electrocatalytic NO_3^-RR performance of R-$Co_1Cu_\delta O_X$ (δ = 0, 5, 10, 15, 20, 50, simplified stoichiometric ratio representation, R-CuO_X, R-$Co_1Cu_{19}O_X$, R-$Co_1Cu_9O_X$, R-$Co_3Cu_{17}O_X$, R-$Co_1Cu_4O_X$, R-$Co_1Cu_1O_X$) composites with different feeding molar ratios.** (a) LSV curves at a scan rate of 10 mV s^{-1} in 1 M KOH solution with and without 100 mM NO_3^-; (b) NH_3 yield rate; (c) FEs of NH_3; and (d) the comparison of NH_3 yield rate and FEs of NH_3 after 3600 s electrolysis at -0.2V vs. RHE (The error bars (mean ± standard deviation) are obtained based on three independent electro-catalytic experiments).

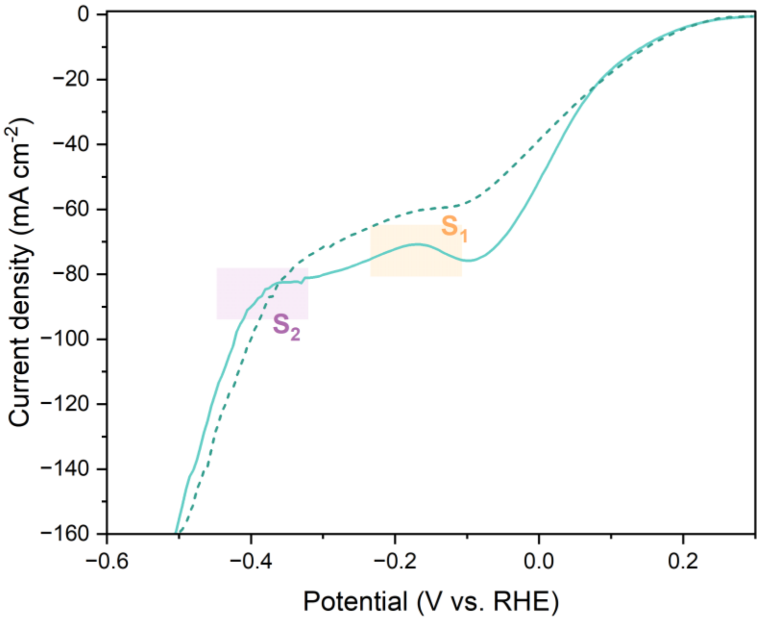

Figure S24. Cyclic voltammograms obtained for R-$Co_1Cu_9O_X$ in 1.0 M KOH with 100 mM NO_3^- at a scan rate of 10 mV s^{-1}.

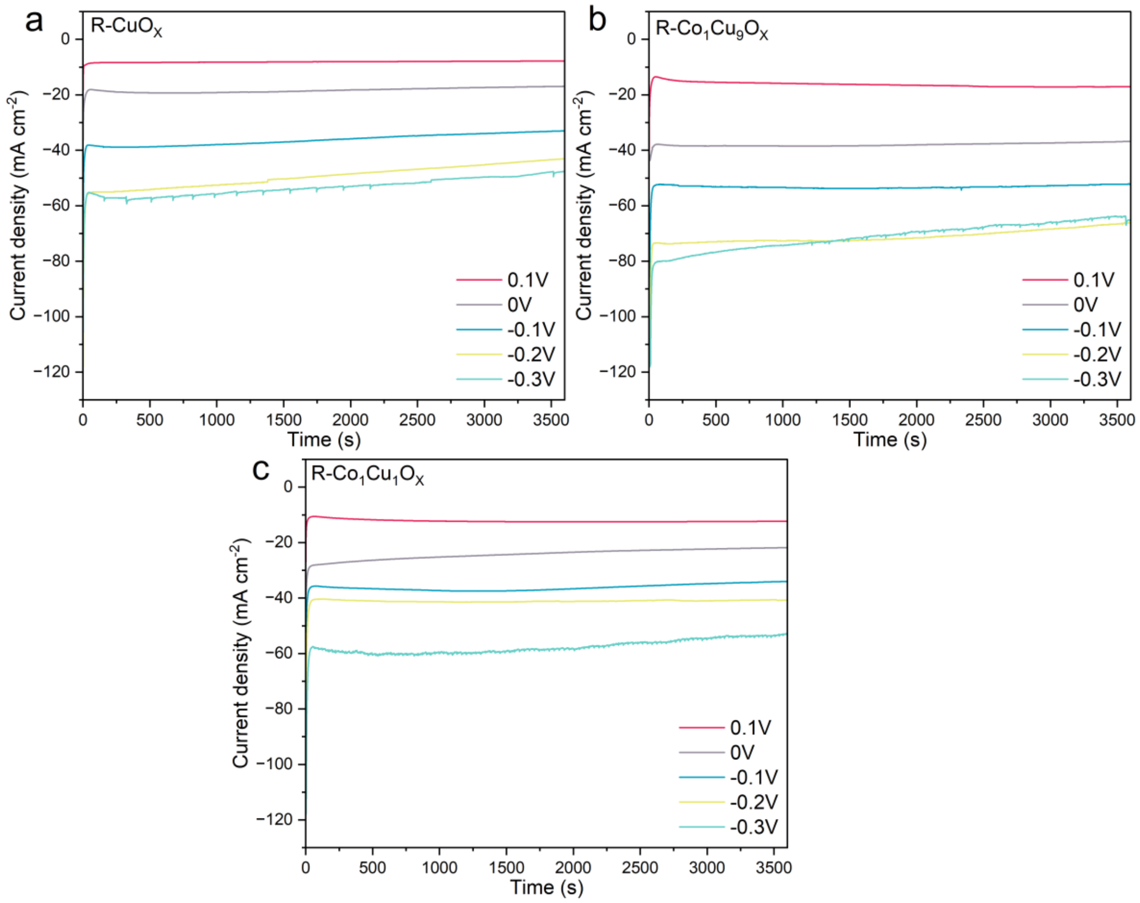

Figure S25. Plot of i–t characteristics for (a) R-CuO$_X$, (b) R-Co$_1$Cu$_9$O$_X$, (c) R-Co$_1$Cu$_1$O$_X$ catalysts at various potentials in 1 M KOH with 100 mM NO_3^-.

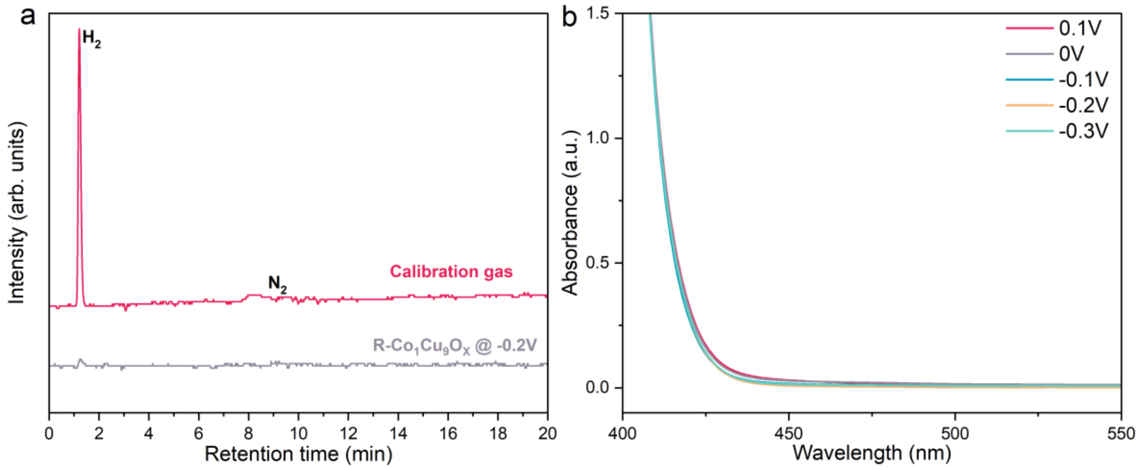

Figure S26. (a) Gas chromatography test for R-$Co_1Cu_9O_X$ at −0.2 V vs. RHE. (b) UV-Vis absorption spectra of N_2H_4 with R-$Co_1Cu_9O_X$ after electrolysis at different potentials.

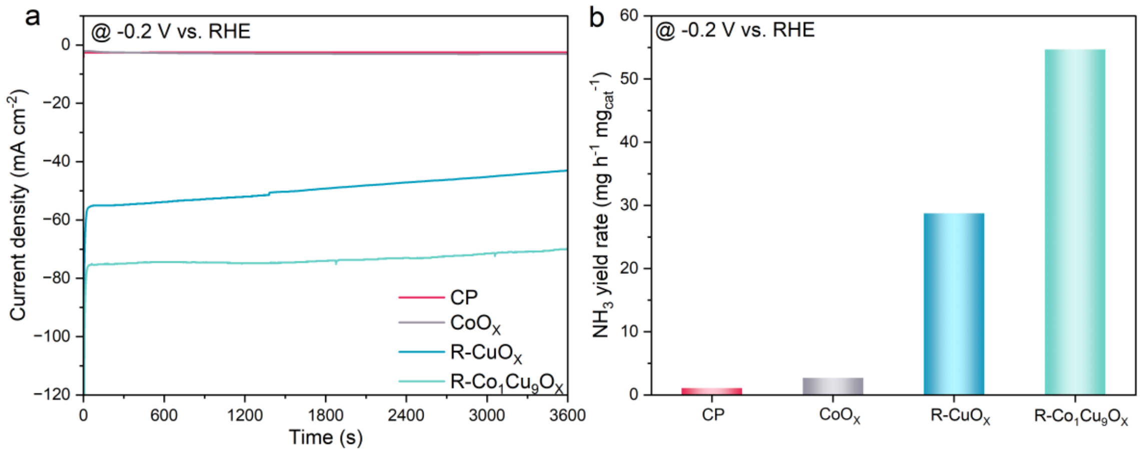

Figure S27. Comparison of (a) i–t curves and (b) NH_3 yield rate at -0.2 V vs. RHE in 1 M KOH solution with 100 mM NO_3^- of bare carbon paper (CP), CoO_X, R-CuO_X, and R-$Co_1Cu_9O_X$.

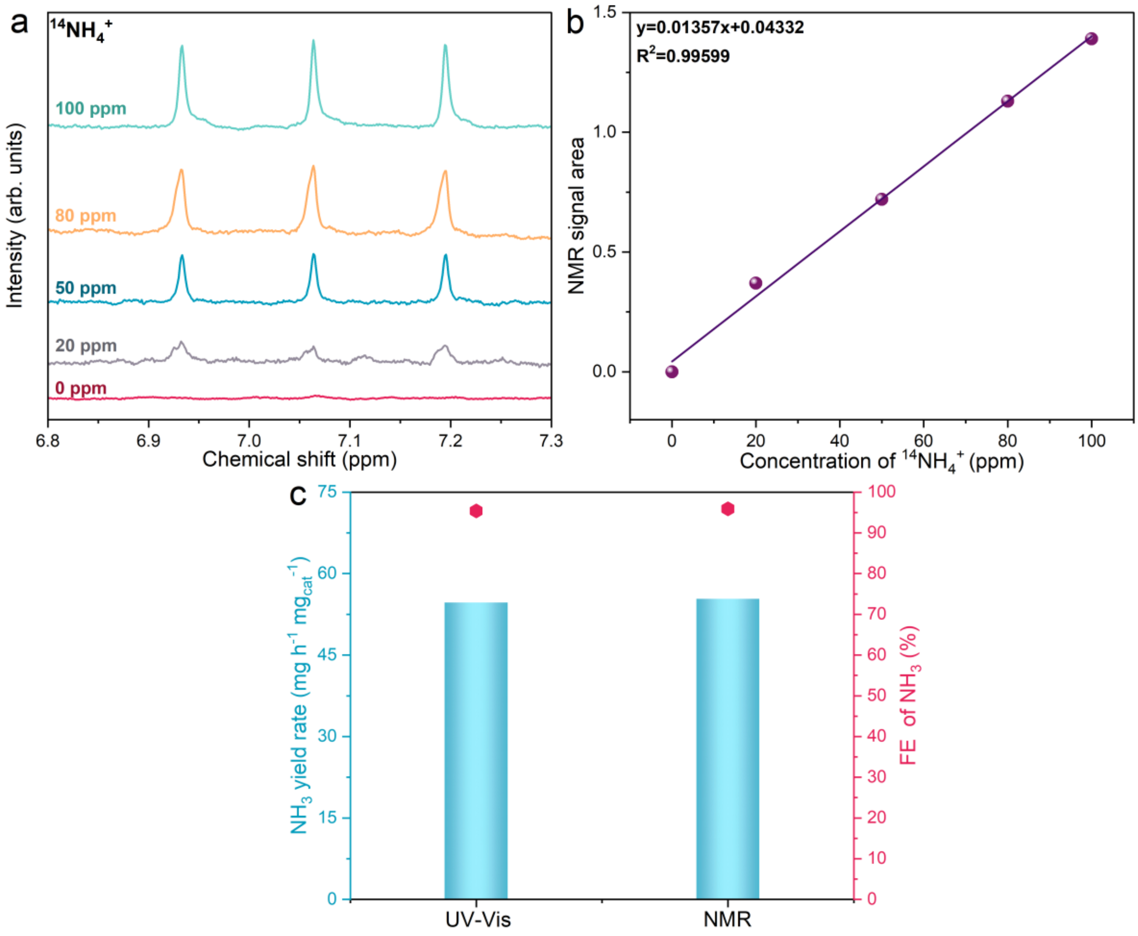

Figure S28. (a) ^{1}H NMR spectra of standard $^{14}NH_4^+$ samples. (b) Calibration curve of the ^{1}H NMR signal from standard $^{14}NH_4^+$ samples. (c) Comparison of NH_3 yield rate and NH_3 FE of R-$Co_1Cu_9O_X$ determined by UV-Vis method and ^{1}H NMR measurement.

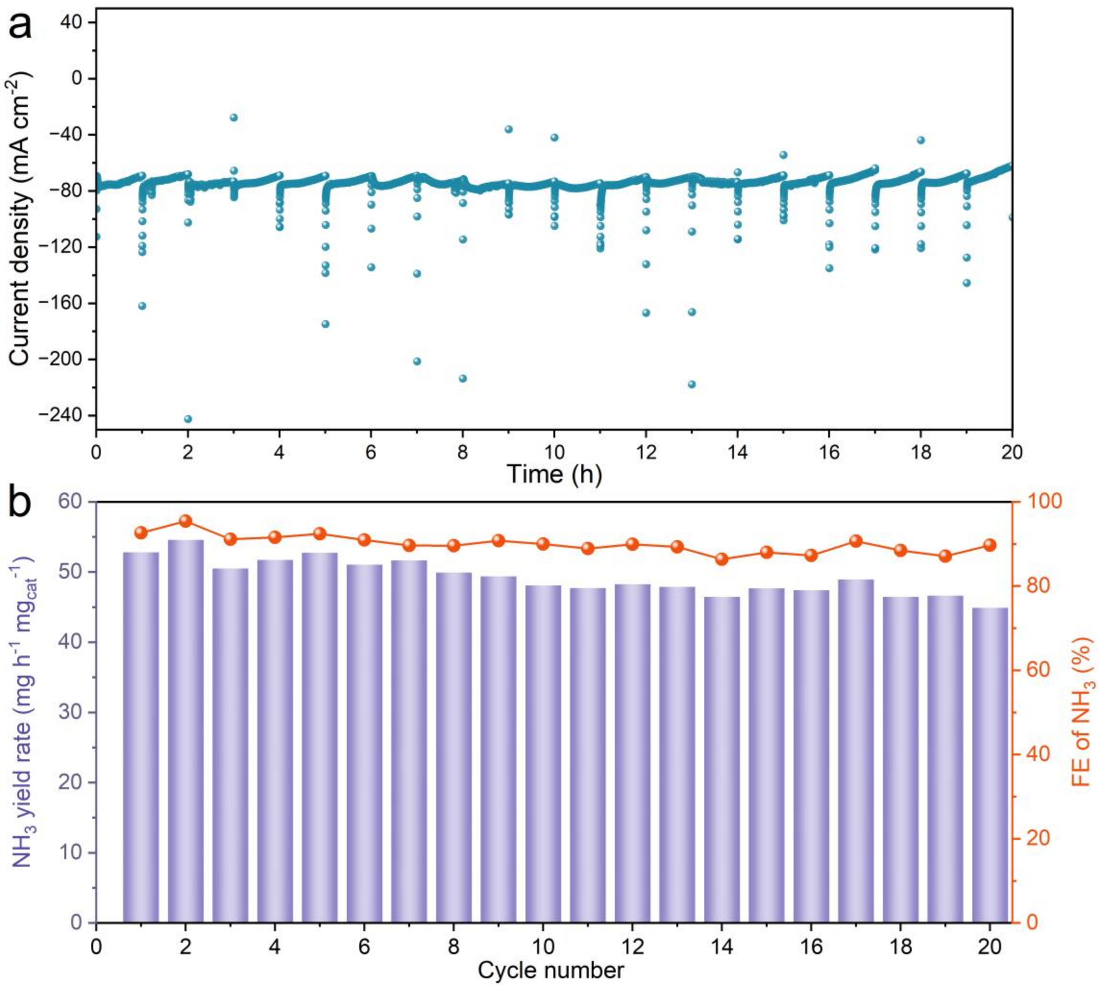

Figure S29. Stability test results for R-$Co_1Cu_9O_X$ at -0.2 V vs. RHE during 20 h NO_3^-RR in a H-type reactor. (b) Stability measurement of R-$Co_1Cu_9O_X$ during cyclic reduction tests at −0.2 V vs. RHE.

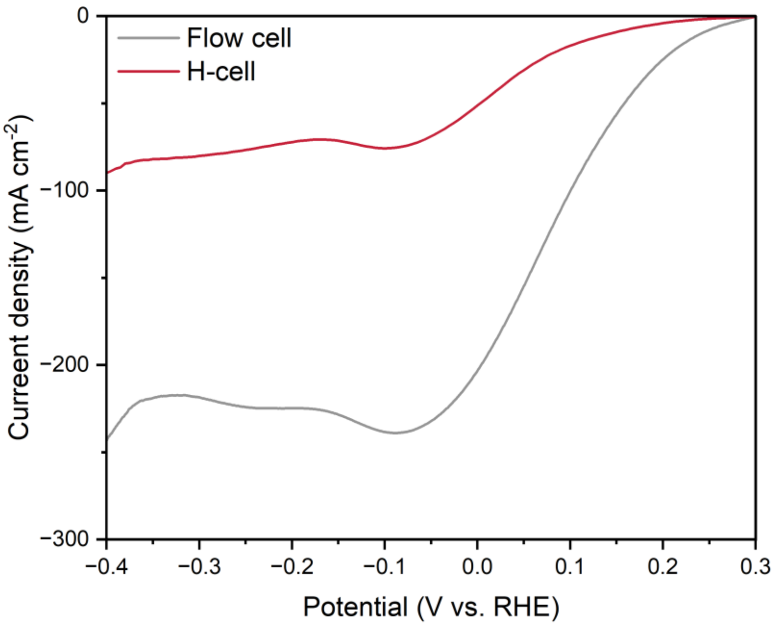

Figure S30. The comparison of LSV plots on the R-$Co_1Cu_9O_X$ catalyst in H-type reactor and flow-cell configurations.

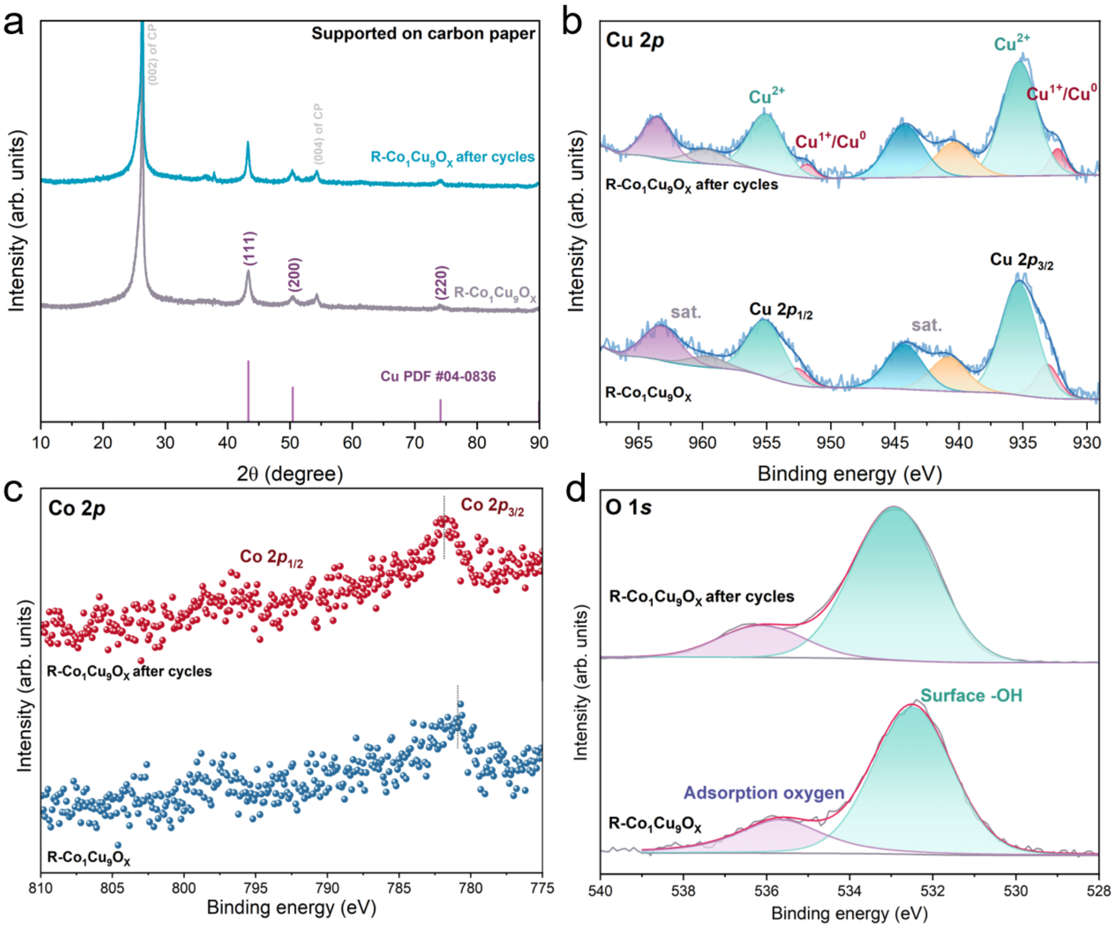

Figure S31. (a) XRD pattern and XPS spectra of (b) Cu 2*p*, (c) Co 2*p*, and (d) O 1*s* of R-$Co_1Cu_9O_X$ after stability test.

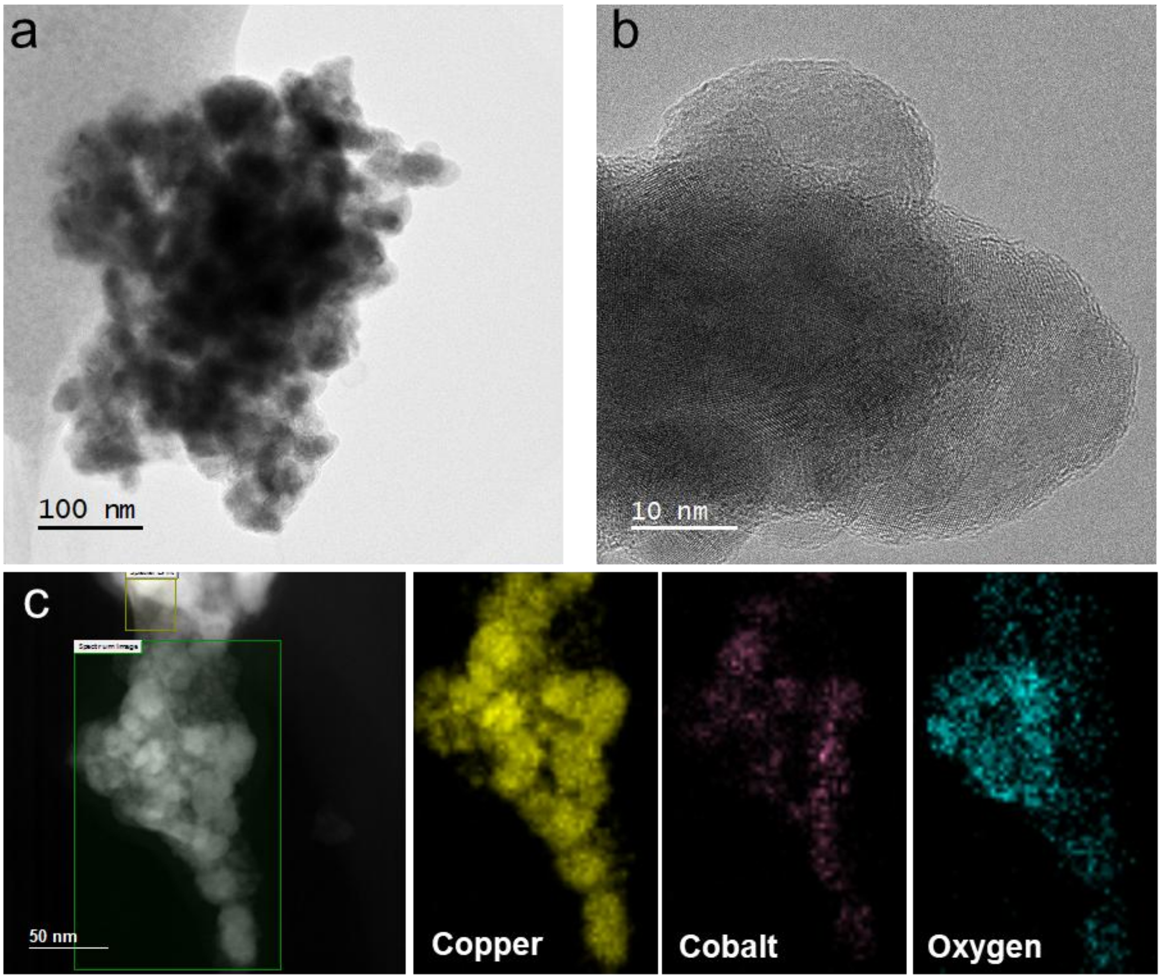

Figure S32. (a) TEM, (b) HR-TEM, and (c) STEM images along with the corresponding EDS element map of R-$Co_1Cu_9O_X$ after stability test.

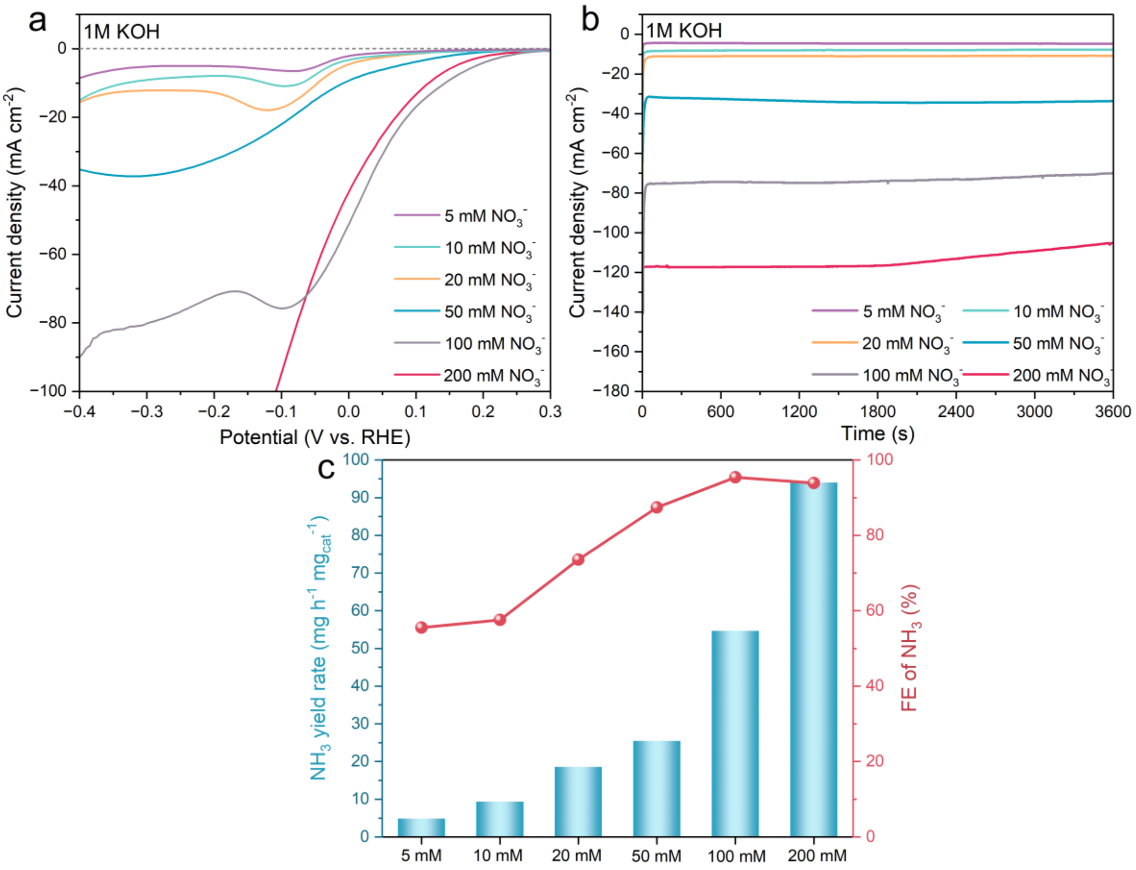

Figure S33. (a) LSV curves at a scan rate of 10 mV s^{-1}. (b) Chronoamperometry curves of R-$Co_1Cu_9O_X$ at -0.2 V vs. RHE for 3600 s in 1.0 M KOH solution containing different NO_3^- concentrations. (c) Comparison of NO_3^- RR performance of R-$Co_1Cu_9O_X$ under different NO_3^- concentrations.

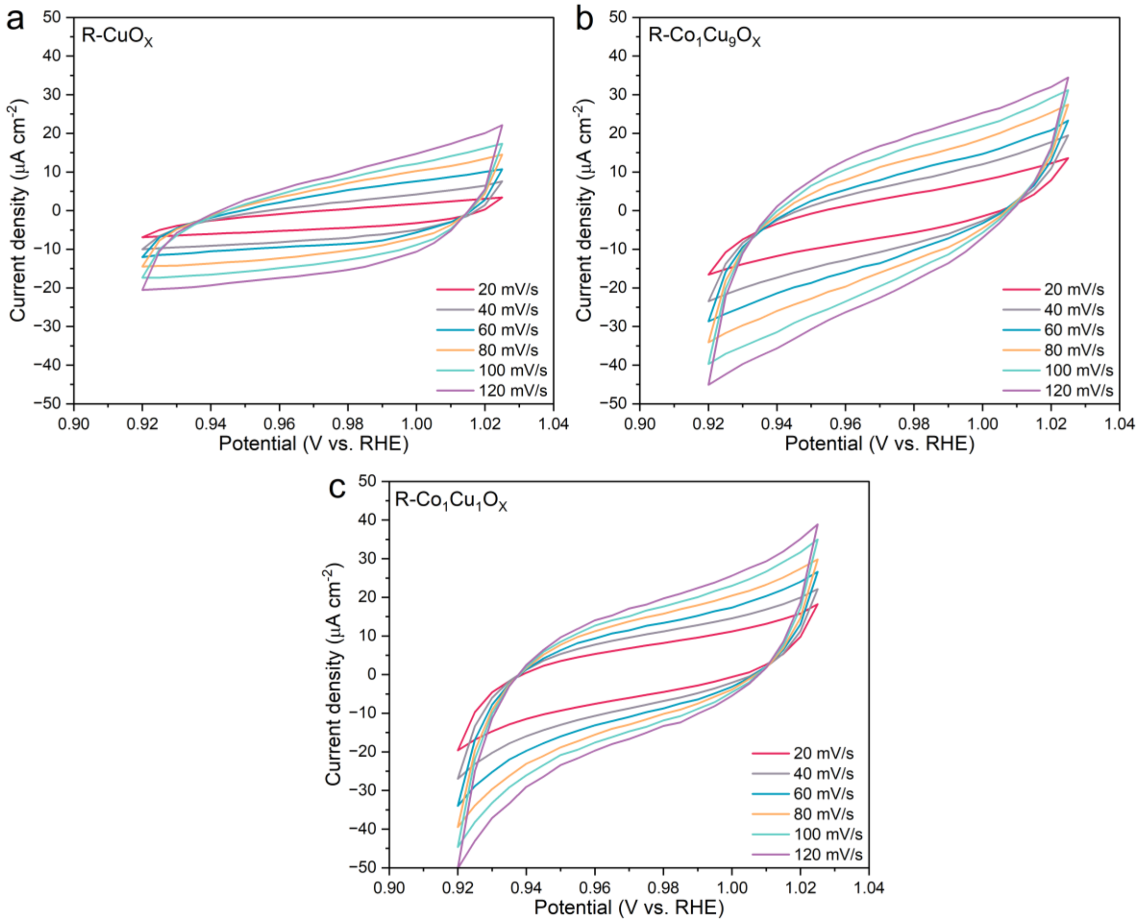

Figure S34. Cyclic voltammograms of (a) R-CuO_X, (b) R-$Co_1Cu_9O_X$, and (c) R-$Co_1Cu_1O_X$ in the non-Faraday zone at different scan rates.

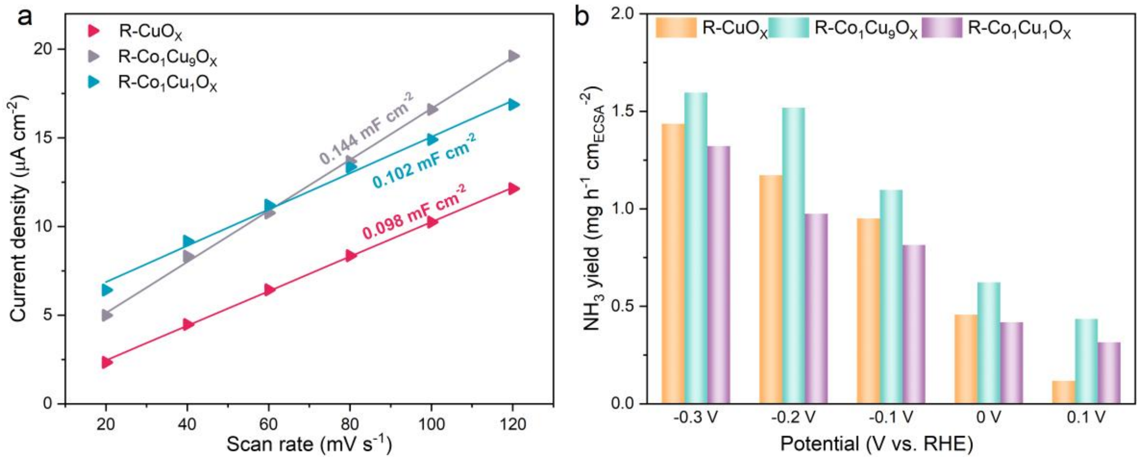

Figure S35. (a) C_{dl} values and (b) NH_3 yield rates of R-CuO_X, R-$Co_1Cu_9O_X$, and R-$Co_1Cu_1O_X$ normalized to ECSA.

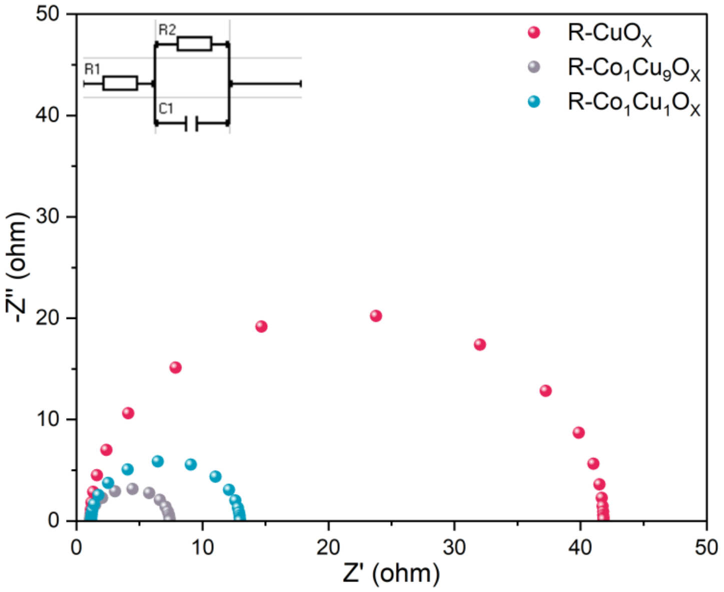

Figure S36. Electrochemical impedance spectra of R-CuO_X, R-$Co_1Cu_9O_X$, and R-$Co_1Cu_1O_X$ catalysts.

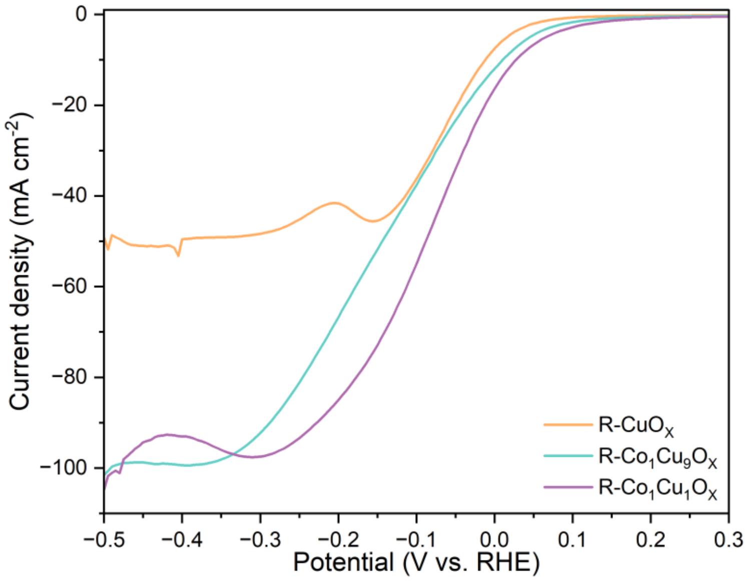

Figure S37. LSV curves of R-CuO_X, R-$Co_1Cu_9O_X$, and R-$Co_1Cu_1O_X$ catalysts at a scan rate of 10 mV s^{-1} in 1 M KOH solution with 100 mM NO_2^-.

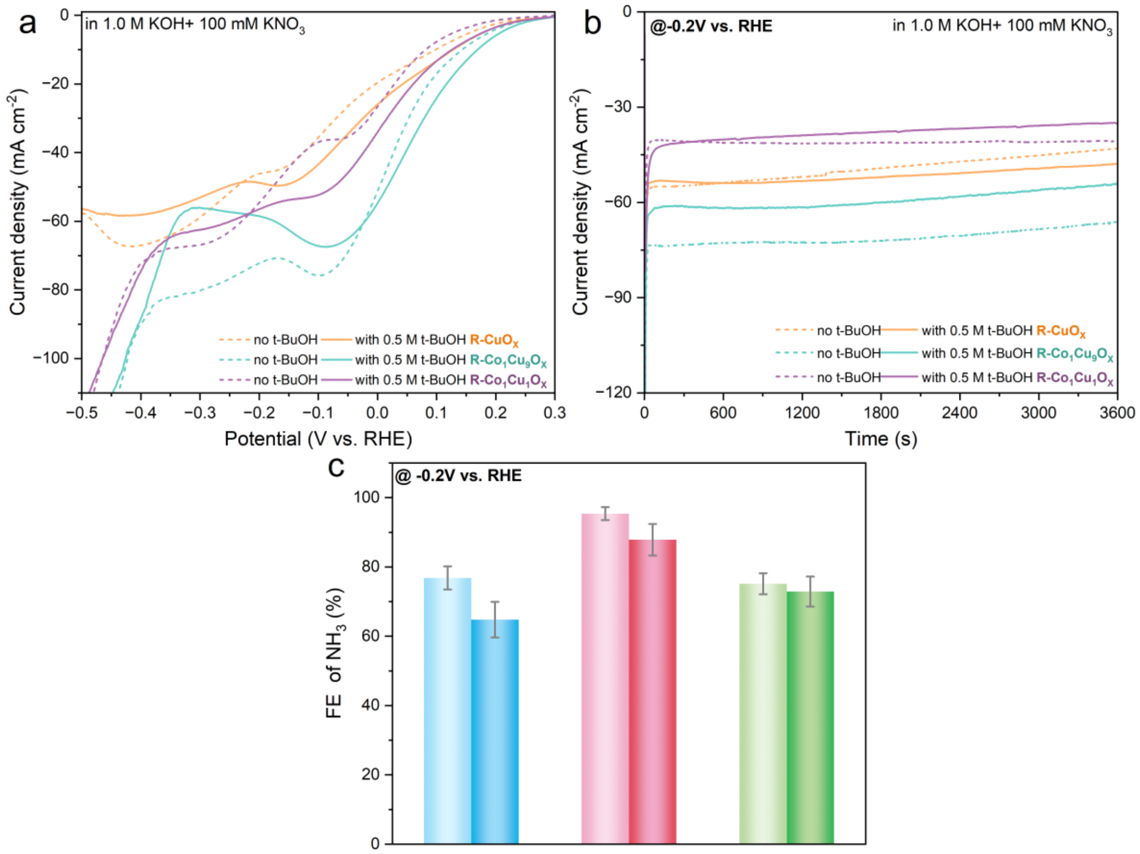

Figure S38. (a) LSV curves; (b) it curves at -0.2V vs. RHE; (c) NH_3 FE of R-CuO_X, R-$Co_1Cu_9O_X$, and R-$Co_1Cu_1O_X$ with and without 0.5 M t-BuOH in 1.0 M KOH + 100 mM KNO_3.

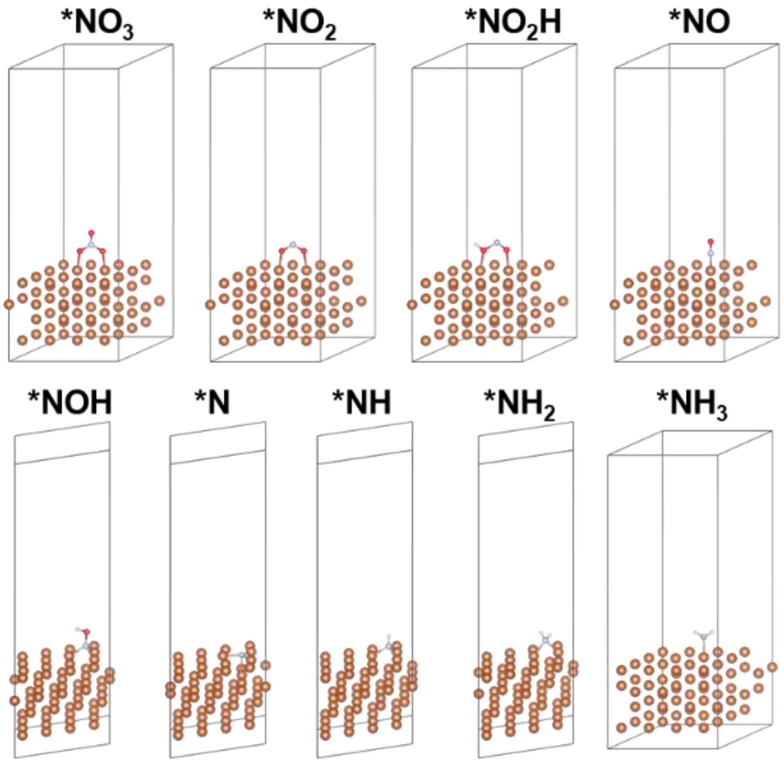

Figure S39. Optimized adsorption configurations of reaction intermediates during NO_3^-RR on Cu (111); Color code: brown for Cu, red for O, light blue for N, light pink for H.

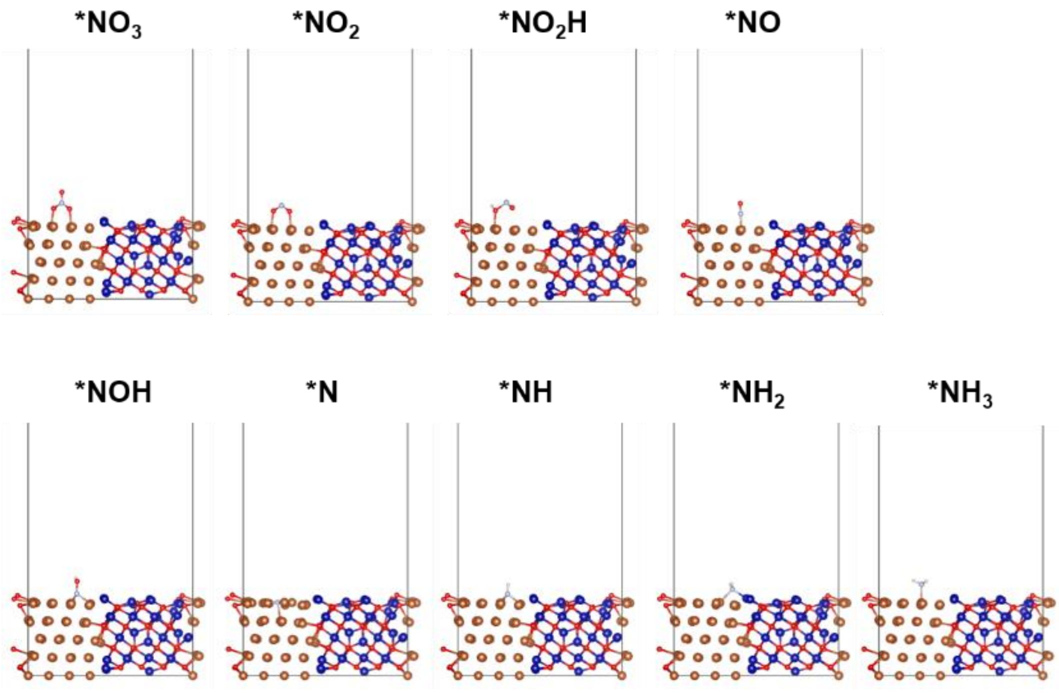

Figure S40. Optimized adsorption configurations of reaction intermediates during NO_3^-RR of Cu(111)/CoO_X. Color code: brown for Cu, blue for Co, red for O, light blue for N, light pink for H.

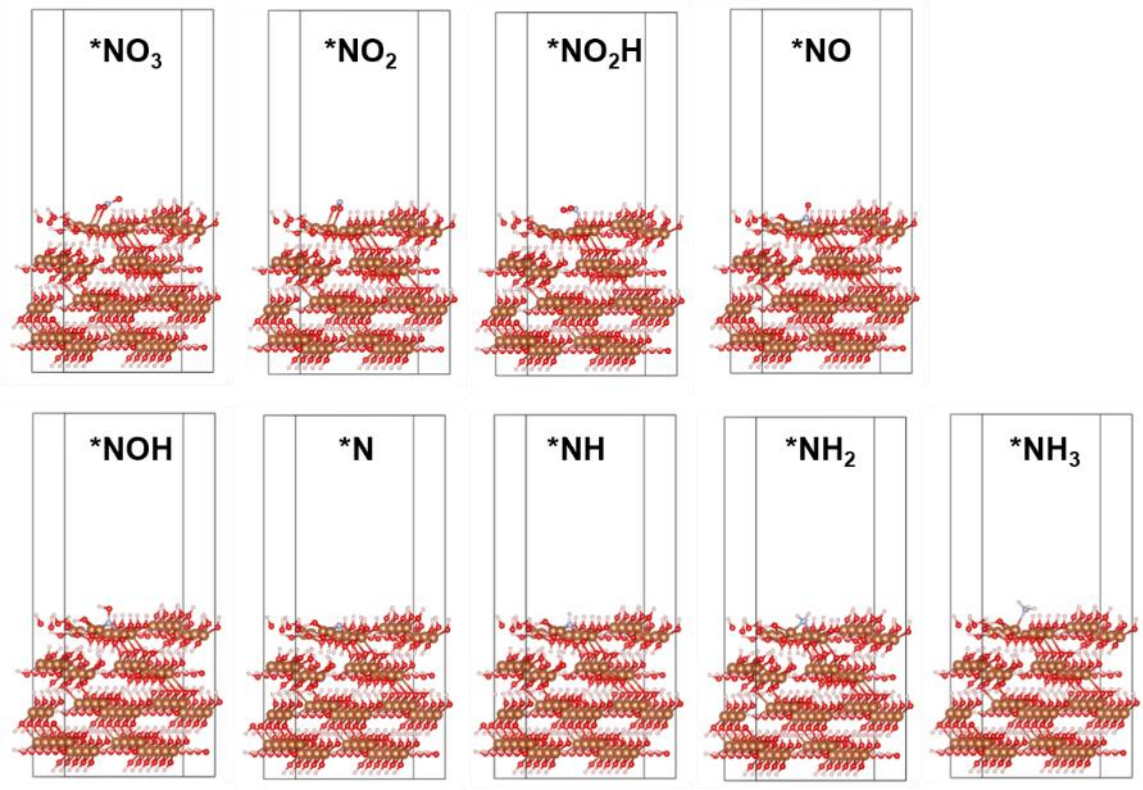

Figure S41. Optimized adsorption configurations of reaction intermediates during NO_3^-RR of hydroxylated oxidized Cu. Color code: brown for Cu, red for O, light blue for N, light pink for H.

Table S1. Comparison of electrochemical NO_3^-RR performance of R-CuO_X and R-$Co_1Cu_9O_X$ catalyst with other reported Cu-based electrocatalysts.

**Catalyst**	**Electrolyte**	**Potential (V vs. RHE)**	**Mass activity (mg h^{-1} mg$_{cat}$$^{-1}$)**	**FE (%)**	**Ref.**
Oxygen vacancy-rich CuO_X	0.1 M KOH + 50 ppm KNO_3	-0.25	0.45	74.18	[11]
Cu-doped Fe_3O_4	0.1 M KOH +100 mM KNO_3	-0.6	179.55	nearly 100%	[12]
Cu/Pd/CuOx	0.5 M K_2SO_4 +50 mg L^{-1} KNO_3	-0.88	1.5103	84.04	[13]
Cu_XO/N-GDY	1 M KOH+100 mM KNO_3	-0.5	0.34	85	[14]
Co-Cu_2O/Cu@C	0.1 m KOH +50 mM KNO_3	-0.4	37.86	98.1	[15]
Cu/a-CeOx	1 M KOH+50 mM KNO_3	-0.5	27.36	98.76	[16]
$Mg_{0.2}Co_{0.2}Ni_{0.2}Cu_{0.2}Zn_{0.2}O$	1 M KOH +500 mM KNO_3	-0.4	26.6	97.2	[17]
Cu/CuxO/graphdiyne(GDY)	1.0 M KOH+100 mM KNO_3	-0.8	25.4	99.8	[18]
CuO NTs	0.5 M K_2SO_4, 50	-0.88	0.7786	85.7	[19]

	mg L^{-1} NO_3^- -N				
$Cu@Cu_{2+1}O$ NWs	0.5 M K_2SO_4, 50 mg L^{-1} NO_3^- -N	-1.2 V vs. SCE	0.57653	87.07	[20]
Cu nanosheets	0.1 M KOH +10 mM KNO_3	-0.15 V	0.3901	99.7	[21]
Pd-Cu_2O CEO	0.5 M K_2SO_4, 50 mg L^{-1} NO_3^- -N	-1.3 V vs. SCE	0.92511	96.56	[22]
Plasma treated Cu_2O	0.5 M K_2SO_4, 50 mg L^{-1} NO_3^- -N	-1.1 V vs. Ag/AgCl	1.494 (0.083 mmol h^{-1} mg^{-1})	89.54	[23]
Nanoporous Cu−Co	1 M KOH +1400 mM KNO_3	-0.23 V	37.656	91.5	[2]
R-CuO_X	1 M KOH +100mM KNO_3	-0.2 V	28.72	76.84	**This work**
R-$Co_1Cu_9O_X$	1 M KOH +100mM KNO_3	-0.2 V	54.68	95.40	**This work**

References:

1. Y. Liu, J. Wei, Z. Yang, et al., "Efficient Tandem Electroreduction of Nitrate into Ammonia Through Coupling Cu Single Atoms with Adjacent Co_3O_4," *Nature Communications* 15 (2024): 3619, https://doi.org/10.1038/s41467-024-48035-4.
2. X. Zhou, W. Xu, Y. Liang, et al., "Dynamically Restructuring Nanoporous Cu–Co Electrocatalyst for Efficient Nitrate Electroreduction to Ammonia," *ACS Catalysis* 14 (2024): 12251–12259, https://doi.org/10.1021/acscatal.4c03336.
3. Y. Cui, C. Sun, G. Ding, et al., "Synergistically Tuning Intermediate Adsorption and Promoting Water Dissociation to Facilitate Electrocatalytic Nitrate Reduction to Ammonia over Nanoporous Ru-doped Cu Catalyst," *Science China Materials* 66 (2023): 4387–4395, https://doi.org/10.1007/s40843-023-2582-6.
4. Y. Cui, A. Dong, Y. Zhou, et al., "Interfacially Engineered Nanoporous Cu/MnOx Hybrids for Highly Efficient Electrochemical Ammonia Synthesis via Nitrate Reduction," *Small* 19 (2023): 2207661, https://doi.org/10.1002/smll.202207661.
5. X. Chen, Y. He, G. Ding, et al., "Ag-$Ni(OH)_2$/Cu Heterostructure for Tandem Electrocatalytic Nitrate Reduction to Ammonia Coupled with 5-Hydroxymethylfurfural Electrooxidation," *Nano Letters* 25 (2025): 9508–9515, https://doi.org/10.1021/acs.nanolett.5c02281.
6. G. Kresse, J. Hafner, "Norm-conserving and Ultrasoft Pseudopotentials for First-row and Transition Elements," *Journal of Physics: Condensed Matter* 6 (1994): 8245, https://doi.org/10.1088/0953-8984/6/40/015.
7. P. E. Blöchl, "Projector Augmented-wave Method," *Physical Review B* 50 (1994): 17953, https://doi.org/10.1103/PhysRevB.50.17953.
8. J. P. Perdew, K. Burke, M. Ernzerhof, "Generalized Gradient Approximation Made Simple," *Physical Review Letters* 77 (1996): 3865–3868, https://doi.org/10.1103/PhysRevLett.77.3865.
9. S. Grimme, J. Antony, S. Ehrlich, H. Krieg, "A Consistent and Accurate ab initio Parametrization of Density Functional Dispersion Correction (DFT-D) for the 94 Elements H-Pu," *The Journal of Chemical Physics* 132 (2010): 4104, https://doi.org/10.1063/1.3382344.
10. S. Grimme, S. Ehrlich, L. Goerigk, "Effect of the Damping Function in Dispersion Corrected Density Functional Theory," *Journal of Computational Chemistry* 32 (2011): 1456–1465, https://doi.org/10.1002/jcc.21759.
11. J. Geng, S. Ji, H. Xu, C. Zhao, S. Zhang, H. Zhang, "Electrochemical Reduction of Nitrate to Ammonia in a Fluidized Electrocatalysis System with Oxygen Vacancy-

rich CuOx Nanoparticles," *Inorganic Chemistry Frontiers* 8 (2021): 5209–5213, https://doi.org/10.1039/D1QI01062J.

12. H. Yin, F. Dong, Y. Wang, et al., "Understanding the Activity Trends in Electrocatalytic Nitrate Reduction to Ammonia on Cu Catalysts," *Nano Letters* 23 (2023): 11899–11906, https://doi.org/10.1021/acs.nanolett.3c03962.
13. T. Ren, Z. Yu, H. Yu, et al., "Interfacial Polarization in Metal-organic Framework Reconstructed Cu/Pd/CuO_x Multi-phase Heterostructures for Electrocatalytic Nitrate Reduction to Ammonia," *Applied Catalysis B: Environmental* 318 (2022): 121805, https://doi.org/10.1016/j.apcatb.2022.121805.
14. J. Li, R. Valenza, S. Haussener, "In situ Synthesis of Cu_xO/N doped Graphdiyne with Pyridine N Configuration for Ammonia Production via Nitrate Reduction," *Small* 20 (2024): 2310467, https://doi.org/10.1002/smll.202310467.
15. Y. Qu, T. Dai, Y. Cui, et al., "Heterostructured Co-doped-Cu_2O/Cu Synergistically Promotes Water Dissociation for Improved Electrochemical Nitrate Reduction to Ammonia," *Small* 20 (2024): 2308246, https://doi.org/10.1002/smll.202308246.
16. Y. Li, C. Wang, L. Yang, et al., "Enhancement of Nitrate-to-Ammonia on Amorphous CeO_x-modified Cu via Tuning of Active Hydrogen Supply," *Advanced Energy Materials* 14 (2024): 2303863, https://doi.org/10.1002/aenm.202303863.
17. S. Sun, C. Dai, P. Zhao, et al., "Spin-Related Cu-Co Pair to Increase Electrochemical Ammonia Generation on High-Entropy Oxides," *Nature Communications* 15 (2024): 260, https://doi.org/10.1038/s41467-023-44587-z.
18. X. Feng, J. Liu, Y. Kong, et al., "Cu/CuxO/graphdiyne Tandem Catalyst for Efficient Electrocatalytic Nitrate Reduction to Ammonia," *Advanced Materials* 36 (2024): 2405660, https://doi.org/10.1002/adma.202405660.
19. C. Li, S. Liu, Y. Xu, et al., "Controllable Reconstruction of Copper Nanowires into Nanotubes for Efficient Electrocatalytic Nitrate Conversion into Ammonia," *Nanoscale* 14 (2022): 12332–12338, https://doi.org/10.1039/D2NR03767J.
20. T. Ren, K. Ren, M. Wang, et al., "Concave-convex Surface Oxide Layers over Copper Nanowires Boost Electrochemical Nitrate-to-ammonia Conversion," *Chemical Engineering Journal* 426 (2021): 130759, https://doi.org/10.1016/j.cej.2021.130759.
21. X. Fu, X. Zhao, X. Hu, et al., "Alternative Route for Electrochemical Ammonia Synthesis by Reduction of Nitrate on Copper Nanosheets," *Applied Materials Today* 19 (2020): 100620, https://doi.org/10.1016/j.apmt.2020.100620.
22. Y. Xu, K. Ren, T. Ren, et al., "Ultralow-content Pd In-situ Incorporation Mediated Hierarchical Defects in Corner-etched Cu_2O Octahedra for Enhanced

Electrocatalytic Nitrate Reduction to Ammonia," *Applied Catalysis B: Environmental* 306 (2022): 121094, https://doi.org/10.1016/j.apcatb.2022.121094.

23. Z. Gong, W. Zhong, Z. He, et al., "Regulating Surface Oxygen Species on Copper (I) Oxides via Plasma Treatment for Effective Reduction of Nitrate to Ammonia," *Applied Catalysis B: Environmental* 305 (2022): 121021, https://doi.org/10.1016/j.apcatb.2021.121021.
